# Gut microbiome dysbiosis in antibiotic-treated COVID-19 patients is associated with microbial translocation and bacteremia

**DOI:** 10.1038/s41467-022-33395-6

**Published:** 2022-11-01

**Authors:** Lucie Bernard-Raichon, Mericien Venzon, Jon Klein, Jordan E. Axelrad, Chenzhen Zhang, Alexis P. Sullivan, Grant A. Hussey, Arnau Casanovas-Massana, Maria G. Noval, Ana M. Valero-Jimenez, Juan Gago, Gregory Putzel, Alejandro Pironti, Evan Wilder, Yale IMPACT Research Team, Abeer Obaid, Alice Lu-Culligan, Allison Nelson, Anderson Brito, Angela Nunez, Anjelica Martin, Annie Watkins, Bertie Geng, Chaney Kalinich, Christina Harden, Codruta Todeasa, Cole Jensen, Daniel Kim, David McDonald, Denise Shepard, Edward Courchaine, Elizabeth B. White, Eric Song, Erin Silva, Eriko Kudo, Giuseppe DeIuliis, Harold Rahming, Hong-Jai Park, Irene Matos, Jessica Nouws, Jordan Valdez, Joseph Fauver, Joseph Lim, Kadi-Ann Rose, Kelly Anastasio, Kristina Brower, Laura Glick, Lokesh Sharma, Lorenzo Sewanan, Lynda Knaggs, Maksym Minasyan, Maria Batsu, Mary Petrone, Maxine Kuang, Maura Nakahata, Melissa Campbell, Melissa Linehan, Michael H. Askenase, Michael Simonov, Mikhail Smolgovsky, Nicole Sonnert, Nida Naushad, Pavithra Vijayakumar, Rick Martinello, Rupak Datta, Ryan Handoko, Santos Bermejo, Sarah Prophet, Sean Bickerton, Sofia Velazquez, Tara Alpert, Tyler Rice, William Khoury-Hanold, Xiaohua Peng, Yexin Yang, Yiyun Cao, Yvette Strong, Lorna E. Thorpe, Dan R. Littman, Meike Dittmann, Kenneth A. Stapleford, Bo Shopsin, Victor J. Torres, Albert I. Ko, Akiko Iwasaki, Ken Cadwell, Jonas Schluter

**Affiliations:** 1 Kimmel Center for Biology and Medicine at the Skirball Institute, New York University Grossman School of Medicine, New York, NY USA; 2 Vilcek Institute of Graduate Biomedical Sciences, New York University Grossman School of Medicine, New York, NY USA; 3 Department of Immunobiology, Yale School of Medicine, New Haven, CT USA; 4 Division of Gastroenterology, Department of Medicine, New York University Grossman School of Medicine, New York, NY USA; 5 Institute for Systems Genetics, New York University Grossman School of Medicine, New York, NY USA; 6 Department of Epidemiology of Microbial Diseases, Yale School of Public Health, New Haven, CT USA; 7 Department of Microbiology, New York University Grossman School of Medicine, New York, NY USA; 8 Department of Population Health, New York University Grossman School of Medicine, New York, NY USA; 9 Antimicrobial-Resistant Pathogens Program, New York University School of Medicine, New York, NY USA; 10 Howard Hughes Medical Institute, Chevy Chase, MD USA; 11 Department of Medicine, Division of Infectious Diseases, New York University Grossman School of Medicine, New York, NY USA; 12 Yale School of Medicine, New Haven, CT USA; 13 Department of Molecular Biophysics and Biochemistry, Yale School of Medicine, New Haven, CT USA; 14 Department of Medicine, Section of Pulmonary and Critical Care Medicine, Yale School of Medicine, New Haven, CT USA; 15 Yale Viral Hepatitis Program, Yale School of Medicine, New Haven, CT USA; 16 Yale Center for Clinical Investigation, Yale School of Medicine, New Haven, CT USA; 17 Department of Medicine, Section of Infectious Diseases, Yale School of Medicine, New Haven, CT USA; 18 Department of Neurology, Yale School of Medicine, New Haven, CT USA; 19 Department of Molecular, Cellular and Developmental Biology, Yale School of Medicine, New Haven, CT USA

**Keywords:** Microbiome, Applied microbiology

## Abstract

Although microbial populations in the gut microbiome are associated with COVID-19 severity, a causal impact on patient health has not been established. Here we provide evidence that gut microbiome dysbiosis is associated with translocation of bacteria into the blood during COVID-19, causing life-threatening secondary infections. We first demonstrate SARS-CoV-2 infection induces gut microbiome dysbiosis in mice, which correlated with alterations to Paneth cells and goblet cells, and markers of barrier permeability. Samples collected from 96 COVID-19 patients at two different clinical sites also revealed substantial gut microbiome dysbiosis, including blooms of opportunistic pathogenic bacterial genera known to include antimicrobial-resistant species. Analysis of blood culture results testing for secondary microbial bloodstream infections with paired microbiome data indicates that bacteria may translocate from the gut into the systemic circulation of COVID-19 patients. These results are consistent with a direct role for gut microbiome dysbiosis in enabling dangerous secondary infections during COVID-19.

## Abstract (web summary)

Here, the authors show that SARS-CoV-2 infection causes gut microbiome dysbiosis and gut epithelial cell alterations in a mouse model, and correlate dysbiosis observed in COVID-19 patients with blood stream infections, matching reads of bacterial sequences from stool samples to organisms found in the blood.

## Introduction

A better understanding of factors contributing to the pathology of coronavirus disease 2019 (COVID-19) is an urgent global priority. Previous reports have demonstrated that severe COVID-19 is frequently associated with specific inflammatory immune phenotypes, lymphopenia, and a generally disproportionate immune response leading to systemic organ failure^1,2^. Even in mild cases, gastrointestinal symptoms are reported frequently, and recent studies reported that COVID-19 patients lose commensal taxa of the gut microbiome during hospitalization^3–5^, and persistent microbiome alterations are found in patients with long-term complications from COVID-19^6–8^. Differences in gut bacterial populations relative to healthy controls were observed in all COVID-19 patients, but most strongly in patients who were treated with antibiotics during their hospitalization^4^. Most recently, COVID-19 patients treated with broad-spectrum antibiotics at admission were shown to have increased susceptibility to multi-drug resistant infections and nearly double the mortality rate from septic shock^9,10^. Furthermore, although initially estimated to be low (6.5%)^11^, more recent studies have detected bacterial secondary infections in as much as 12–14% of COVID-19 patients^12–14^. However, the causal direction of the relationship between disease symptoms and gut bacterial populations is not yet clear.

Complex gut microbiota ecosystems can prevent the invasion of potentially pathogenic bacteria^15,16^. Conversely, when the gut microbiota incurs damage, such as through antibiotics treatment, competitive exclusion of pathogens is weakened^17–19^ and potentially dangerous blooms of antibiotic-resistant bacterial strains can occur^20,21^. In immunocompromised cancer patients, blooms of Enterococcaceae and Gram-negative proteobacteria can lead to gut dominations by few or single species^22–25^. Such gut domination events are dangerous to these patients because they are associated with increased risk of translocation of antibiotic-resistant bacteria from the gut into the blood stream^22,26,27^. Bacterial co-infection can also cause life-threatening complications in patients with severe viral infections^10,11,28^; therefore, antibacterial agents were administered empirically to nearly all critically ill suspected COVID-19 patients since the incidence of bacterial superinfection was unknown early during the pandemic^4,29^. However, it is now known that nosocomial infection during prolonged hospitalization is the primary threat to patients with COVID-19^30^, rather than bacterial co-infection upon hospital admission^12,31–33^. Evidence from immunocompromised cancer patients suggests that indiscriminate administration of broad-spectrum antibiotics may, counter-intuitively, increase nosocomial bloodstream infection (nBSI) rates by causing gut dominations of resistant microbes that can translocate into the blood^22,34^. Indeed, we recently showed that *Enterococcus*, a common gut microbial genus comprised of intrinsically antibiotic-resistant strains, accounts for a large proportion of nBSIs during longer hospitalizations, suggesting gut translocation^35^. Thus, empiric antimicrobial use, i.e., without direct evidence for a bacterial infection, in patients with severe COVID-19 may be especially pernicious because it can select for antimicrobial resistance and promote gut translocation-associated nBSI.

The role of the gut microbiome in respiratory viral infections in general^36–39^, and in COVID-19 patients, in particular, is only beginning to be understood. Animal models of influenza virus infection have uncovered mechanisms by which the microbiome influences antiviral immunity^40–42^, and in turn, the viral infection was shown to disrupt the intestinal barrier of mice by damaging the gut microbiota^43,44^. Here, we show that infection by SARS-CoV-2 alone causes gut microbiome dysbiosis and gut epithelial cell alterations in a mouse model. We analyze stool samples obtained from two independent cohorts of patients at NYU Langone Health and Yale New Haven Hospital, and find that COVID-19 is associated with severe microbiome injury characterized by loss of diversity and anaerobe taxa, resembling observations made in the mouse model. Analysis of sequencing reads obtained from stool samples together with results from blood cultures, we find that gut dysbiosis in COVID-19 patients is associated with secondary bloodstream infections by gut bacteria.

## Results

### Gut microbiome dysbiosis in SARS-CoV-2-infected mice

**Fig. 1:**
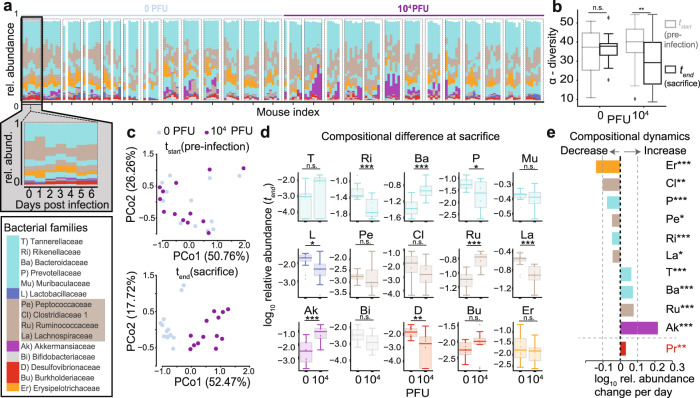
SARS-CoV-2 infection causes gut microbiome alterations in mice. K18-hACE2 mice were infected intranasally with 0 or 10^4^ PFU of SARS-CoV-2. Fecal samples for microbiome analyses were collected daily from day 0 (before infection) until sacrifice; mice were sacrificed on days 5–7. Results show pooled data from three independent experiments with *n* = 3–5 mice per group. **a** Timelines of fecal microbiota composition measured by 16S rRNA gene sequencing. Bars represent the composition of the 15 most abundant bacterial families per sample for each day, blocks of samples correspond to an individual mouse’s time course from day 0 to day 6, as exemplified for the first mouse. **b** *α*-diversity (inverse Simpson index) per infection group in the beginning (*t*_*start*_, *n* = 13 each for control and infected) and at the end (*t*_*end*_, *n* = 13 each for control and infected) of the experiment (n.s.: non-significant, **: *p* < 0.01, one-tailed, paired *t*-test; boxplots show median and quartile ranges). Comparison between infected and non-infected mouse microbiomes at the end of the experiment. **c** Principal coordinate plot of bacterial compositions in samples collected prior to infection (*t*_start_, top) and at sacrifice (*t*_end_, bottom) of the experiment (Bray Curtis dissimilarity). **d** *log*_10_-relative family abundances at the final time point; boxplots show median and quartile ranges, whiskers extend to 1.5 times max- and min- quartile values, n.s.: not significant; *: *p* value < 0.05; **: *p* value < 0.01; ***: *p* value < 0.001; two-sided Wilcoxon rank-sum tests (*n* = 13 each for control and infected). **e** Analysis of microbiome composition trajectories in infected mice. Regression coefficients of the estimated changes in family abundances per day in mice infected with 10^4^ PFU were obtained from linear mixed effects models with varying effects per mouse and per cage (only significant coefficient results shown, abbreviations and colors as per the bacterial family legend; Red: separate, analogous analysis for phylum Proteobacteria trajectories).

We first determined whether SARS-CoV-2 infection could directly cause gut dysbiosis independently of hospitalization and treatment. K18-hACE2 mice (*K18-ACE2tg* mice), express human *ACE2* driven by the *cytokeratin-18* promoter (*K18-ACE2tg* mice). Although the overexpression of ACE2 prevents investigation of long-term consequences of infection due to potential non-specific disease, which is a major limitation of the model, an advantage of these mice is that they develop severe respiratory disease in a virus dose-dependent manner, partially mirroring what is observed in COVID-19 patients^45–48^. Daily changes in fecal bacterial populations were monitored following intranasal inoculation of mice with a range of doses (10, 100, 1000, and 10^4^ PFU) of SARS-CoV-2 or mock-treatment (Fig. 1a, Supplementary Fig. 1). Although we detected viral RNA in the lungs but not in the intestine or stool as previously observed^49^ of mice infected with doses as low as 100 PFU (Supplementary Fig. 1c), mice inoculated with doses lower than 10^4^ PFU displayed minimal or no signs of disease (Supplementary Fig. 1a, b). As expected, based on this outcome, shifts in their microbiome were inconsistent (Supplementary Fig. 2a–d). Thus, we focused on findings from the 10^4^ PFU inoculum.

Mice infected with 10^4^ PFU displayed weight loss and other signs of disease around day 4 (Supplementary Figs. 1a, b, 2h, i), alongside microbiome changes characterized by a significant loss of alpha diversity (inverse Simpson index, Fig. 1b) corresponding to shifts in the bacterial community composition (Fig. 1c, d). We performed time series analyses on bacterial family abundances, contrasting their trajectories in infected (10^4^ PFU) and uninfected mice. This revealed that the strongest shift over time in infected mice was characterized by significant increases of Akkermansiaceae (*p* < 0.0002, Fig. 1d). Ranking all bacterial family trajectories by their estimated changes over time in infected mice showed that this increase in Akkermansiaceae was accompanied by significant losses of Clostridiaceae 1, a family of obligate anaerobe bacteria, and of Erysipelotrichiaceae (Fig. 1e). We also performed a trajectory analysis on the abundance of Proteobacteria, a phylum that comprises many pathogenic taxa that are a major cause of nBSIs^22^; we observed a significant increase of Proteobacteria over time in infected mice (Fig. 1e), but not in uninfected mice (Supplementary Fig. 2e). This increase of Proteobacteria in infected mice was driven by increase in several proteobacterial genera, with the steepest increase observed in *Escherichia/Shigella* (Supplementary Fig. 2f, g). These results demonstrated that SARS-CoV-2 infection leads to gut microbiome dysbiosis over time in a mouse model.

We then determined if this dysbiosis was also associated with intestinal defects that could enable translocation of bacteria into the blood. In mice infected with 1000 PFU, bacterial translocation in spleen and liver was observed in more of the infected mice compared to uninfected controls (Supplementary Fig. 3a). However, while several of the mice infected with 10^4^ PFU displayed signs of barrier dysfunction, the observed differences in plasma concentrations of fluorescein isothiocyanate (FITC)-dextran following its administration by gavage, or other markers of intestinal barrier permeability, fatty acid-binding protein, lipopolysaccharide-binding protein, and citrulline did not reach significance (Supplementary Fig. 3b, c). The reduced colon lengths, as well as reductions in the villus lengths in the duodenum or ileum, i.e., markers of overt inflammation, that we observed, were also non-significant compared with control mice (Supplementary Fig. 3d, e). Interestingly, infected mice that had incurred the most severe microbiome injury in the form of diversity loss also showed the most evidence of gut permeability—the highest FITC-dextran concentrations in the blood of mice detected across all samples came from the mice with the most extreme dysbiosis and highest levels of Akkermansiaceae, a family of mucin-degrading bacterial species (Supplementary Fig. 4).

### SARS-CoV-2 infection alters gut epithelium in mice

**Fig. 2:**
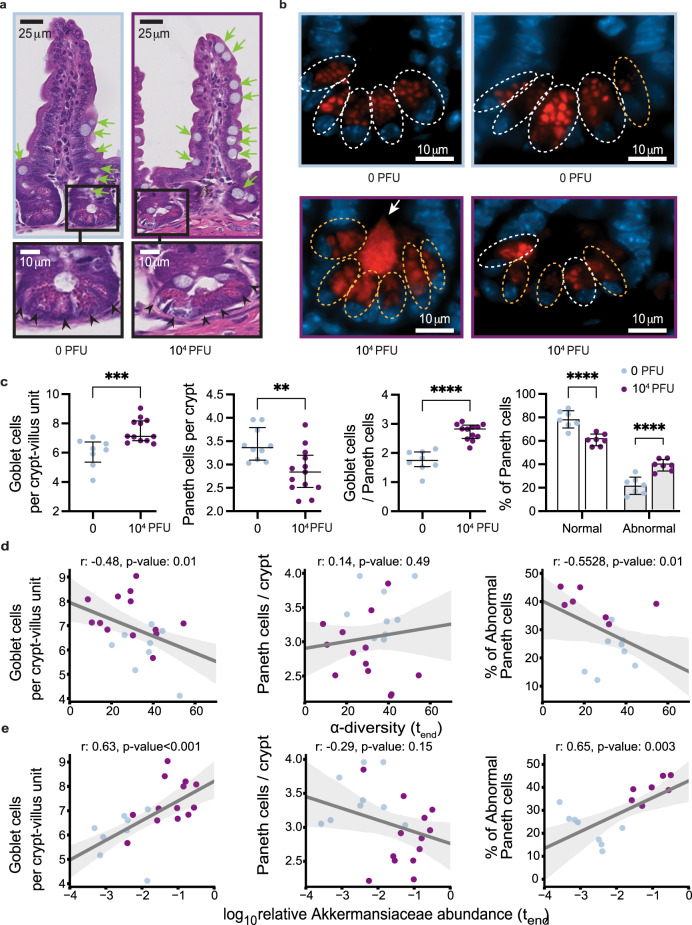
SARS-CoV-2 infection causes abnormalities in the gut epithelium of mice. K18-hACE2 were inoculated intranasally with 10^4^ PFU SARS-CoV-2 or mock treatment. **a** Representative H&E-stained section of the ileum depicting crypt-villus axes from mice at the end of the experiment. Green arrows indicate goblet cells, scale bars correspond to 25 μm. Bottom panels show high magnification images of the indicated crypt with black arrowheads pointing at Paneth cells, scale bars correspond to 10 μm. **b** Representative anti-lysozyme immunofluorescence images of the ileal crypt (two images per group). White and orange dotted circles delineate normal and abnormal Paneth cells, respectively. Abnormality is characterized by distorted, depleted, or diffuse lysozyme distribution patterns in Paneth cells. Lysozyme = red, DAPI = blue, scale bars correspond to 10 μm. **c** Quantification of goblet cell number per villus (left), Paneth cells per crypt (middle left) and ratio of goblet cell number / Paneth cell number (middle right) based on H&E staining, and frequency of normal versus abnormal Paneth cell lysozyme distribution pattern based on the immunofluorescence staining as depicted in **b** (**right**). Dots represent the mean cell number per crypt-villus unit in each mouse, 50 units were counted per mouse. Results were pooled from three independent experiments with *n* = 3–5 mice per group for each experiment (*n* = 8–14 control mice, 12–14 infected mice). Some mice were excluded from the analysis when quality of the slides was too poor. Boxplots indicate median and interquartile ranges (ns = non-significant, *p* < 0.05; **, *p* < 0.01; ***, *p* < 0.001; ****, *p* < 0.0001 two-sided Mann-Whitney *U* test). **d** Correlation of Goblet cell number per villus (left, two-sided Pearson correlation *r* = −0.48, *p* = 0.015), Paneth cells per crypt (middle, *r* = 0.14, *p* value = 0.483) and frequency of abnormal Paneth cell lysozyme distribution pattern (right, *r* = −0.5528, *p* = 0.014) for the mice shown in **c** with α-diversity (inverse Simpson) of the gut microbiome measured at the last day before sacrifice. e Correlation of Goblet cell number per villus (left, *r* = 0.63, *p* < 0.001), Paneth cells per crypt (middle, *r* = −0.29, *p* = 0.149) and frequency of abnormal Paneth cell lysozyme distribution pattern (right, *r* = 0.65, *p* value = 0.003) for the mice shown in **c** with log_10_-relative abundances of *Akkermansia* in fecal samples from the last day before sacrifice; lines: univariate linear regression, shaded region: 95% CI.

Interestingly, we also detected a significant increase in the number of mucus-producing goblet cells and a decrease in the number of Paneth cells in the ileum (but not in the duodenum) of infected mice (Fig. 2a, c and Supplementary Fig. 3f). The decrease in Paneth cells was accompanied by structural abnormalities, most notably deformed or misplaced granules (Fig. 2b), and reduced gene expression of several antimicrobial factors such as lysozyme, defensins, Reg3γ and serum amyloid A in the ileum (Supplementary Fig. 3g). These morphological abnormalities in Paneth cells were reminiscent of observations in the ileum of patients with inflammatory bowel disease (IBD) as well as in a virally-triggered animal model of IBD, where such structures were indicative of defects in packaging and secretion of the granule protein lysozyme^50–52^. Thus, to quantify the Paneth cell granule defect, we performed lysozyme immunofluorescence and found a significant increase in the proportion of Paneth cells displaying abnormal staining patterns compared with the controls (Fig. 2b, c). We then investigated if these physiological defects were associated with dysbiosis in the microbiome.

The most severely sick mice also had the most striking shifts in their microbiome composition and the lowest microbiota diversity at the end of the experiment (Supplementary Fig. 4a, b). To associate the observed physiological defects with microbiome dysbiosis, we plotted the numbers of goblet cells per crypt-villus unit and Paneth cells per crypt, as well as the percentage of abnormal Paneth cells against bacterial alpha diversity and the log_10_-relative abundance of Akkermansiaceae (Fig. 2d, e). Goblet cell counts per crypt-villus unit were negatively correlated with alpha diversity, and, conversely, positively correlated with Akkermansiaceae. No statistically significant association was found between diversity, Akkermansiaceae abundance, and Paneth cell counts per crypt. However, we observed a striking significant positive correlation between the percentage of abnormal Paneth cells and Akkermansiaceae, and a corresponding negative correlation with diversity. Altogether, these results show that the gut microbiome dysbiosis observed in K18-hACE2 mice infected with a high dose of SARS-CoV-2 is associated with alterations in key epithelial cells, and signs of barrier permeability in the mice displaying the greatest disruption in microbiome diversity.

### Dysbiotic microbiomes and BSIs in COVID-19 patients

**Fig. 3:**
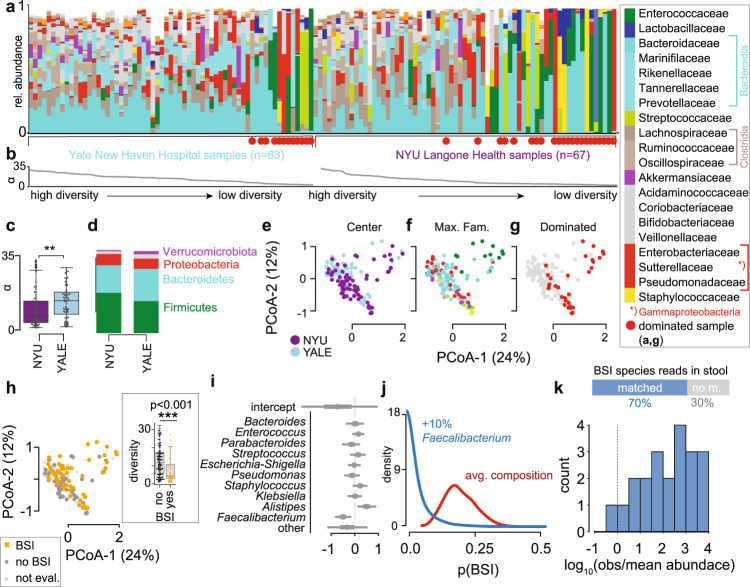
The dysbiotic gut microbiome in COVID-19 in patients from NYU Langone Health (*n* = 60) and Yale New Haven Hospital (*n* = 36) is associated with secondary bloodstream infections. **a** Bacterial family composition in stool samples (Yale, *n* = 63 samples; NYU, *n* = 67) identified by 16S rRNA gene sequencing; bars represent the relative abundances of bacterial families; red circles indicate samples with single taxa >50%. Samples are sorted by center and bacterial *α*-diversity (inverse Simpson index, **b**). **c** *α*-diversity in samples from NYU Langone Health and Yale New Haven Hospital; *p* = 0.0065, two-sided T-test. **d** Average phylum level composition per center. Principal coordinate plots of all samples shown in a, labeled by center (**e**), most abundant bacterial family (**f**) and domination status of the sample (**g**), and BSI status; inset: boxplot of inverse Simpson index diversity by BSI (**h**). **i** Coefficients from a Bayesian logistic regression with most abundant bacterial genera as predictors of BSI status (circle: posterior mean, lines: 95% HDI). **j** Counterfactual posterior predictions of BSI risk based on bacterial composition contrasting the predicted risk of the average composition across all samples (red) with the risk predicted from a composition where *Faecalibacterium* was increased by 10% (blue). **k** Shotgun metagenomic reads matched the species identified in clinical blood cultures in 70% of all investigated cases; the histogram shows the distribution of log_10_-ratios of relative abundances of matched species in corresponding stool samples to their corresponding mean abundances across all samples.

To investigate the microbiome in COVID-19 patients, we profiled the bacterial composition of the fecal microbiome in 130 samples (Fig. 3a) obtained from SARS-CoV-2 infected patients treated at NYU Langone Health (NYU, 67 samples from 60 patients) and Yale New Haven Hospital (YALE, 63 samples from 36 patients, Supplementary Table 1). Analysis of metagenomic data obtained from sequencing of the 16S rRNA genes revealed a wide range of bacterial community diversities, as measured by the inverse Simpson index, in samples from both centers (NYU: [1.0, 32.3], YALE: [1.5, 29.3], Fig. 3b); on average, samples from NYU were less diverse (−4, *p* < 0.01, two-tailed*T* test, Fig. 3c), and as reported previously, samples from patients admitted to the ICU had reduced diversity (−3.9, *p* < 0.05, two-tailed*T* test, Supplementary Fig. 5a). However, the composition in samples between the two centers did not show systematic compositional differences (Fig. 3d–f). On average, in both centers, members of the phyla Firmicutes and Bacteroidetes represented the most abundant bacteria, followed by Proteobacteria (Fig. 3d). The wide range of bacterial diversities was reflected in the high variability of bacterial compositions across samples (Fig. 3e, f). In samples from both centers, microbiome dominations, defined as a community where a single genus reached more than 50% of the population, were observed frequently (NYU: 21 samples, YALE: 12 samples), representing states of severe microbiome injury in COVID-19 patients (Fig. 3g, Supplementary Fig. 5). Strikingly, samples associated with a BSI, defined here as a positive clinical blood culture test result, had strongly reduced bacterial α-diversities (mean difference: −5.2, CI_BEST_[−8.2, −2.2], Fig. 3h).

The lower diversity associated with samples from 25 patients with BSIs (26% of the patients, 15 NYU, 10 Yale, Supplementary Table 2) led us to investigate their bacterial taxon compositions and the potential that gut dysbiosis was associated with BSI events. Importantly, BSI patients had received antibiotic treatments during hospitalization (Supplementary Fig. 6, Supplementary Table 2), which could exacerbate COVID-19-induced shifts in microbiota populations^20,21,24^, and may indeed be administered in response to a suspected or confirmed BSI. We noted that most BSI patients received antibiotics prior to their BSI, with 6 out of 25 patients receiving antibiotics only after the detection of BSI. Principal coordinate analysis of all stool samples indicated that the BSI-associated samples spanned a broad range of compositions (Fig. 3h). To identify bacterial abundance patterns that consistently distinguished BSI from non-BSI-associated samples, we performed a Bayesian logistic regression. The model estimated the association of the ten most abundant bacterial genera with BSI cases, i.e., it identified enrichment or depletion of bacterial genera in BSI associated samples (Fig. 3i). This analysis revealed that the genus *Faecalibacterium* was negatively associated with BSI (OR: −0.5, CI:[−0.86, −0.15]), which was also observed when we included microbiome domination as an additional factor in the model (Supplementary Fig. 7a). However, our analysis also included stool samples that were taken only after a positive blood culture was obtained, calling into question the plausibility of gut translocation; a complementary analysis only using stool samples obtained prior or on the same day of a positive blood culture also identified *Faecalibacterium* as most negatively associated with BSI (Supplementary Fig. 7b). Furthermore, a higher-resolution analysis using amplicon sequencing variant (ASV) relative abundances as predictors of BSI (Supplementary Fig. 7c, d), identified an ASV of the *Faecalibacterium* genus as most negatively associated with BSI, in agreement with our main analysis. *Faecalibacterium* is an immunosupportive, short-chain fatty acid-producing genus that is a prominent member of the human gut microbiome^53–55^, and its reduction is associated with disruption to intestinal barrier function^56,57^, perhaps via ecological network effects^57^.

To evaluate the effect size of the association between *Faecalibacterium* and BSIs, we performed a counterfactual posterior predictive check. Using the average genus composition found across all samples, we first computed the distribution of predicted BSI risks (Fig. 3j), and compared this risk distribution with a hypothetical bacterial composition which increased *Faecalibacterium* by 10% points. The predicted risk distributions associated with these two compositions differed strongly (mean difference 15%, CI: [1%, 32%], Fig. 3j). Domination states of the microbiome increase the risk for BSIs in immunocompromised cancer patients^22^; such dominations imply high relative abundances of single taxa, and therefore a low diversity. Consistent with this, *Faecalibacterium* abundance was positively correlated with diversity (R: 0.55, *p* < 10^−10^, Supplementary Fig. 8) in our data set and as reported previously^53^.

We therefore next investigated a direct association between the bacteria populating the gut microbiome and the organisms identified in the blood of patients. Visualizing the bacterial composition in stool samples from patients alongside the BSI microorganisms (Supplementary Fig. 9a) suggested a correspondence with the respective taxa identified in the blood: high abundances of the BSI-causing microbes were found in corresponding stool samples. A rank abundance analysis matching the organisms identified in clinical blood cultures to the composition of bacteria in corresponding stool samples indicated enrichment of taxa belonging to the same bacterial orders as BSI-causing organisms (Supplementary Fig. 9b), suggesting translocation of bacteria from the gut into the bloodstream.

To further investigate evidence for translocation of gut bacteria into the blood, we next performed shotgun metagenomic sequencing on a subset of BSI-associated samples with sufficient remaining material; this allowed us to match the organism identified in clinical blood cultures at the species level with reads obtained from stool samples (Fig. 3k, Supplementary Table 3). In four cases of positive blood cultures of *Staphylococcus* species, no reads matching the clinically identified species were detected (Supplementary Table 3). This may explain why the rank analysis suggested that Staphylococcales were not generally enriched in BSIs by *Staphylococcus* (Supplementary Fig. 9a, b). Consistent with this, in one case of a *S. aureus* BSI where corresponding stool relative abundances of *Staphylococcus* were low, reads from shotgun sequencing did not match the genomes of isolates obtained from the same patient better than *S. aureus* genomes from other isolates (Supplementary Fig. 9d). On the other hand, several reference genomes of *S. aureus* have almost identical sequences, allowing reads from the stool sample to align almost the same extent to all of them. Strikingly, shotgun metagenomic reads matched the genome of isolates well in another case where relative abundances of *Staphylococcus* were enriched in the stool (Supplementary Fig. 9c), providing evidence that here, the same strains were found in stool and blood of the same patient. In all investigated cases of positive blood cultures by organisms other than *Staphylococcus*, the species identified in clinical blood cultures had corresponding reads in the stool samples. Strikingly, the relative abundances of matched species tended to be larger than the average abundances of matched species across all samples (Supplementary Table 3).

## Discussion

Collectively, these results reveal an unappreciated link between SARS-CoV-2 infection, gut microbiome dysbiosis, and a severe complication of COVID-19, BSIs. The loss of diversity and immunosupportive *Faecalibacterium* in patients with BSIs mirrored a similar loss of diversity in the most severely sick mice deliberately infected with SARS-CoV-2, and as observed by other labs and other model systems^58–60^. Notably, a recent study reproduced these changes in the microbiome in an antibiotics-naïve cohort^7^, suggesting that the viral infection causes gut dysbiosis, either through gastrointestinal infection^61–65^ or through a systemic inflammatory response^2,4^. Furthermore, the pronounced increase in Akkermansiaceae in mice was also observed in our patient samples and has been reported previously in patients and in K18-hACE2 mice^58,66^. However, the dysbiosis in patients with COVID-19 exceeded the microbiota shifts observed in the mouse experiments, including microbiome dominations by single taxa, which was not seen in the mouse experiments. It is possible that in our experiment, mice were sacrificed before perturbations to the gut microbial populations reached a maximum. hACE2 knock-in mice, which display reduced disease^45^, were not tested in the scope of this study but could provide additional insights in the future. However, it is also plausible that the frequently administered antibiotic treatments that hospitalized COVID-19 patients receive exacerbated SARS-CoV-2-induced microbiome perturbations. Additionally, unlike the controlled environment experienced by laboratory mice, hospitalized patients are uniquely exposed to antimicrobial-resistant infectious agents present on surfaces and shed by other patients.

Despite these limitations of the mouse model, we observed that SARS-CoV-2 infection led to alteration of intestinal epithelial cells with established roles in intestinal homeostasis and gastrointestinal disease^67,68^. Microbiome ecosystem shifts are likely both cause and consequence of these epithelial cell alterations since epithelial secretions are predicted to affect overall community structure disproportionately strongly^69,70^. For example, disruption of Paneth cell-derived antimicrobials including lysozyme are sufficient to impact microbiome composition^71–73^, and, conversely, *Akkermansia*, which was increased in infected mice, can have epithelium remodeling properties^74^. *Akkermansia* has emerged as a genus of major interest, but its contributions to health or disease are still under research: beneficial health effects^53,75^, as well as detrimental associations, have been reported^76–78^.

Our observation that the type of bacteria that entered the bloodstream was enriched in the associated stool samples is a well-characterized phenomenon in cancer patients^22,26,27^, especially during chemotherapy-induced leukocytopenia when patients are severely immunocompromised^20,53^. COVID-19 patients are also immunocompromised and frequently incur lymphopenia, rendering them susceptible to secondary infections^79^. Our data suggest dynamics in COVID-19 patients may be similar to those observed in cancer patients: BSI-causing organisms may translocate from the gut into the blood, potentially due to loss of gut barrier integrity, through tissue damage downstream of antiviral immunity instead of chemotherapy. Consistent with this possibility, soluble immune mediators such as TNFα and interferons produced during viral infections, including SARS-CoV-2, damage the intestinal epithelium to disrupt the gut barrier, especially when the inflammatory response is sustained as observed in patients with severe COVID-19^52,80,81^. Indeed, blood plasma in severely sick COVID-19 patients is enriched for markers of disrupted barrier integrity and higher levels of inflammation markers^82^, and nBSIs in these patients are often caused by gut microbial taxa^35^, suggesting microbial translocation. Our data support this model with direct evidence because we were able to match sequencing reads from stool samples to genomes of species detected in the blood of patients.

We presented evidence that microorganisms from the dysbiotic gut microbiome translocate into the blood of COVID-19 patients, plausibly due to a combination of the immunocompromising effects of the viral infection and antibiotic-driven depletion of commensal gut microbes. However, COVID-19 patients are also uniquely exposed to other potential factors predisposing them to bacteremia, including immunosuppressive drugs, long hospital stays, and catheters and our study is limited in its ability to investigate their individual effects. Other limitations of our data include the few available whole genome sequences of blood isolates due to discarded blood cultures associated with several BSIs, and the temporal ordering of samples. Occasionally stool samples were collected after observation of BSI, and this mismatch in temporal ordering is counterintuitive for gut-to-blood translocation and a causal interpretation of our associations. However, the reverse direction, that blood infection populates and changes the gut community, is unlikely for the organisms identified in the blood, and if our associations were not causal, we would expect no match between BSI organisms and stool compositions.

Taken together, our findings support a scenario in which gut-to-blood translocation of microorganisms following microbiome dysbiosis leads to dangerous BSIs during COVID-19, a complication seen in other immunocompromised patients, including patients with cancer^22,26,27,83^, acute respiratory distress syndrome^84^, and in ICU patients receiving probiotics^85^. We suggest that investigating the underlying mechanism behind our observations will inform the judicious application of antibiotics and immunosuppressives in patients with respiratory viral infections and increase our resilience to pandemics.

## Methods

### Statistics and reproducibility

No statistical method was used to predetermine sample size. The experiments were not randomized; the investigators were not blinded to allocation during experiments and outcome assessment.

### Mouse experiments

#### Cells and virus

Vero E6 (CRL-1586; American Type Culture Collection) were cultured in Dulbecco’s Modified Eagle’s Medium (Corning) supplemented with 10% fetal bovine serum (Atlanta Biologics) and 1% nonessential amino acids (Corning). SARS-CoV-2, isolate USA-WA1/2020 19 (BEI resources #NR52281), a gift from Dr. Mark Mulligan at the NYU Langone Vaccine Center was amplified once in Vero E6 cells. All experiments with SARS-CoV-2 were conducted in the NYU Grossman School of Medicine ABSL3 facility in accordance with its Biosafety Manual and Standard Operating Procedures, by personnel equipped with powered air-purifying respirators.

#### Mice

Heterozygous K18-hACE2 C57BL/6J mice (strain: 2B6.Cg-Tg(K18-ACE2)2Prlmn/J) were obtained from The Jackson Laboratory. Several were paired with C57BL/6J mice to generate additional heterozygous mice for subsequent experiments and the remaining were used to perform initial experiments. Animals from the same breeder pool (i.e., littermates) were randomly assigned and housed in cages according to the experimental groups in ventilated racks and provided autoclaved water and standard chow ad libitum (dark/light cycle: 12/12 hours, ambient temperature: 69–72 °F, humidity: 30–70%). Cage bedding was mixed prior to infection in a subset of experiments to further reduce possible cage effect. All animal studies were performed according to protocols approved by the NYU School of Medicine Institutional Animal Care and Use Committee (IACUC n°170209 and 180802) and in the ABSL3 facility of NYU Grossman School of Medicine (New York, NY), in accordance with its Biosafety Manual and Standard Operating Procedures. 12-week-old or 24-week-old K18-hACE2 males were administered either 10-10000 PFU SARS-CoV-2 diluted in 50 µL PBS (Corning) or 50 µL PBS (non-infected, 0) via intranasal administration under xylazine-ketamine anesthesia (AnaSedR AKORN Animal Health, KetathesiaTM Henry Schein Inc). Viral titer in the inoculum was verified by plaque assay in Vero E6 cells. Following infection, mice were monitored daily for weight loss, temperature loss and signs of disease. A disease score was calculated as the sum of scores obtained for each of the following criteria: ruffled fur (no = 0, yes = 1), hunched back (no = 0, slightly = 1, exacerbated = 2), heavy breathing (no = 0, yes = 1), altered mobility (no = 1, reduced activity = 1, no mobility = 2). Stool samples were collected and stored at −80 °C.

#### Quantitative real-time PCR to assess viral titer and antimicrobial products

Whole lungs and 1 cm of proximal duodenum, terminal ileum and proximal colon were collected 5 to 7 days after infection. Intestinal pieces were wash with PBS and all organs were transferred in Eppendorf tubes containing 500 μl of PBS and a 5 mm stainless steel bead (Qiagen) and homogenized using the Qiagen TissueLyser II. Homogenates were cleared for 5 min at 5000 × *g*, and the viral supernatant or nasal wash was diluted 4× in TRIzol reagent (Invitrogen) and frozen at −80 °C for titration by qRT-PCR. RNA was extracted from the TRIzol homogenates using chloroform separation and isopropanol precipitation, followed by additional purification using RNeasy spin columns with DNase treatment according to the manufacturer’s instructions (Rneasy Mini Kit; RNAse-Free DNase Set; QIAGEN). RNA was reverse-transcribed using the High-Capacity cDNA Reverse Transcription Kit (Applied Biosystems). To assess viral titer, qPCR was performed using Applied Biosystems TaqMan RNA-to-CT One-Step Kit (Fisher-Scientific), 500 nM of the primers (Fwd 5′-ATGCTGCAATCGTGCTACAA-3′, Rev 5′-GACTGCCGCCTCTGCTC-3′) and 100 nM of the N probe (5′-/56-FAM/TCAAGGAAC/ZEN/AACATTGCCAA/3IABkFQ/−3′). qPCR reaction conditions were 48 °C for 15 min followed by 95 °C for 2 min, and by 50 cycles of: 95 °C for 15 s, and 60 °C for 1 min. Serial dilutions of in vitro transcribed RNA of the SARS-CoV-2 Nucleoprotein (generated as previously described^86^) were used to generate a standard curve and calculate copy numbers per μg of RNA in the samples.

To assess antimicrobial factors, qPCR was performed using SybrGreen (Roche) on a Roche480II Lightcycler using 500 nM of the primers listed in Supplementary Table 4. PCR reaction conditions were 95 °C for 5 min, followed by 45 cycles of: denaturation at 95 °C for 10 s, annealing at 60 °C for 20 s, and extension at 72 °C for 30 s.

#### Microscopy

5 cm of proximal duodenum, distal ileum, and entire colon were flushed with 10% acetate buffered formalin (Fisher scientific), cut open along the length, pinned on black wax and fixed with formalin for 72 h at RT. 2 cm strips of intestinal tissues were embedded in low melting point agarose (Promega) to enrich for well-oriented crypt-villus units. Paraffin embedding, sectioning, and staining were performed by the NYU Experimental Pathology Research Laboratory. 5 μm sections were stained with hematoxylin and eosin and imaged using brightfield wholeslide scanning. Lysozyme staining was performed using anti-lysozyme (ab108508, Abcam) and DAPI immunostaining and analyzed using a Zeiss AxioObserver.Z1 with Axiocam 503 Mono operated with Zen Blue software. 50 small intestinal villi per mouse were measured for villi length. Goblets cell were quantified from 50 villus-crypt units (one villus + half of the 2 surrounding crypts) per mouse. Paneth cells numbers and lysozyme staining patterns were quantified from 50 crypts per mouse. Previously defined criteria were used to quantify the proportion of Paneth cells displaying abnormal lysozyme staining^52^. Mean values were calculated for each mouse and used as individual data points.

#### Measurement of intestinal permeability

Mice were fasted for 4 h before oral gavage with 200 μL of FITC-dextran (3-5 kDa, Sigma-Aldrich) dissolved in sterile PBS (60 mg/ml). After 4 h, mice were euthanized and blood was collected by cardiac puncture. FITC-dextran in plasma was quantified using a plate reader (excitation, 485 nm; emission, 530 nm). Citrulline, intestinal fatty acid-binding protein, and lipopolysaccharide-binding protein were quantified in the plasma by enzyme-linked immunosorbent assay according to the manufacturer’s instructions (MyBioSource, CA). For bacterial translocation assay: spleen and liver were homogenized using the Qiagen TissueLyser II and plated on BBL™ Enterococcosel™ Agar, (BBL, modified esculin bile agar, Becton Dickinson). This medium enriches for enterococci which are often detected following a breach in barrier and will inhibit the growth of most other microorganisms non-specifically present in these organs. Plates were incubated at 37 °C. All colonies were enumerated.

#### Time series analyses of bacterial family abundances

We log_10_-transformed bacterial relative abundances adding a pseudo count to fill zeros (2*10^−6^). We then analyzed the time series with the following model that included fixed effects for the intercepts and slopes of the treatment (i.e., indicator variables for uninfected (0 PFU), and infected (10^4^ PFU), and random effects per cage and per mouse to account for cage effects and repeated measurements from the same individuals, respectively. The model was implemented in the R programming language using the lmerTest v3.1-3.

#### Time series analysis of proteobacterial genus abundances

We altered the model for family abundances to account for sparser genus level abundances by partial pooling data between genera. The genus level model includes a varying intercept and varying slope for each genus, thereby estimating a trajectory for each genus. We only included genera that had non-zero relative abundances in at least five samples; the other proteobacterial genera were under-powered.

### Human study

#### Study population and data collection

This study involved 96 patients with laboratory-confirmed SARS-CoV-2 infection. SARS-CoV-2 infection was confirmed by a positive result of real-time reverse transcriptase-polymerase chain reaction assay on a nasopharyngeal swab. 60 patients were seen at NYU Langone Health, New York, between January 29, 2020–July 2, 2020. In order to be eligible for inclusion in the study, stool specimens needed to be from individuals > 18 years of age. Data including demographic information, clinical outcomes, and laboratory results were extracted from the electronic medical records in the NYU Langone Health clinical management system. Blood and stool samples were collected by hospital staff. OmnigeneGut kits were used on collected stool. In parallel, 36 patients were admitted to YNHH with COVID-19 between 18 March 2020 and 27 May 2020 as part of the YALE IMPACT cohort described at length elsewhere^2^. Briefly, participants were enrolled after providing informed consent and paired blood and stool samples were collected longitudinally where feasible for duration of hospital admission. No statistical methods were used to predetermine sample size for this cohort. Demographic information of patients was aggregated through a systematic and retrospective review of the EHR and was used to construct Supplementary Table 1. Symptom onset and etiology were recorded through standardized interviews with patients or patient surrogates upon enrollment in our study, or alternatively through manual EHR review if no interview was possible owing to clinical status at enrollment. The clinical data were collected using EPIC EHR and REDCap 9.3.6 software. At the time of sample acquisition and processing, investigators were blinded to patient clinical status.

#### DNA extraction and bacterial 16S rRNA gene sequencing

For bacterial DNA extraction 700 µL of SL1 lysis buffer (NucleoSpin Soil kit, Macherey-Nagel) was added to the stool samples and tubes were heated at 95 °C for 2 h to inactivate SARS-CoV-2. Samples were then homogenized using the FastPrep-24TM instrument (MP Biomedicals) and extraction was pursued using the NucleoSpin Soil kit according to the manufacturer’s instructions. DNA concentration was assessed using a NanoDrop spectrophotometer. Samples with too low DNA concentration were excluded. DNA from human samples was extracted with PowerSoil Pro (Qiagen) on the QiaCube HT (Qiagen), using Powerbead Pro (Qiagen) plates with 0.5 mm and 0.1 mm ceramic beads. For mouse samples, the variable region 4 (V4) of the 16S rRNA gene was amplified by PCR using primers containing adapters for MiSeq sequencing and single-index barcodes. All PCR products were analyzed with the Agilent TapeStation for quality control and then pooled equimolar and sequenced directly in the Illumina MiSeq platform using the 2 × 250 bp protocol. Human samples were prepared with a protocol derived from^87^, using KAPA HiFi Polymerase to amplify the V4 region of the 16 S rRNA gene. Libraries were sequenced on an Illumina MiSeq using paired-end 2 × 250 reads and the MiSeq Reagent Kitv2.

#### Bioinformatic processing and taxonomic assignment

Amplicon sequence variants (ASVs) were generated via dada2 v1.16.0 using post-QC FASTQ files. Within the workflow, the paired FASTQ reads were trimmed, and then filtered to remove reads containing Ns, or with maximum expected errors ≥ 2. The dada2 learn error rate model was used to estimate the error profile prior to using the core dada2 algorithm for inferring the sample composition. Forward and reverse reads were merged by overlapping sequence, and chimeras were removed before taxonomic assignment. ASV taxonomy was assigned up to genus level using the SILVAv.138 database with the method described in ref. 88 and a minimum boostrapping support of 50%. Species-level taxonomy was assigned to ASVs only with 100% identity and unambiguous matching to the reference.

#### Shotgun metagenomic sequencing

DNA was quantified with Qiant-iT Picogreen dsDNA Assay (Invitrogen). Libraries were prepared with a procedure adapted from the Nextera Library Prep kit (Illumina), and sequenced on an Illumina NovaSeq using paired-end 2 × 150 reads (Illumina) aiming for 100 M read depth. DNA sequences were filtered for low quality (Q-Score < 30) and length (<50), and adapter sequences were trimmed using cutadapt. Fastq files were converted a single fasta using shi7. Sequences were trimmed to a maximum length of 100 bp prior to alignment. DNA sequences were taxonomically classified using the MetaPhlAn2 v3 analysis tool (http://huttenhower.sph.harvard.edu/metaphlan2). MetaPhlAn2 maps reads to clade-specific marker genes identified from ~17,000 reference genomes and estimates clade abundance within a sample from these mappings.

#### Mapping shotgun reads to whole genome sequences of clinical isolates

Quality-controlled reads were re-classified using Kraken2 (Minikraken2 v2 database, available on https://ccb.jhu.edu/software/kraken2/index.shtml). Reads that were classified by Kraken2 as *Staphylococcus aureus* (or a strain thereof) were further mapped using Bowtie2 separately to each of a collection of *Staphylococcus aureus* isolates. The collection was composed of all NCBI RefSeq assemblies as of 11/17/2021, in addition to *Staphylococcus aureus* isolates that were isolated from our subjects. Bowtie2 mapped reads were then further filtered, keeping only reads that mapped without mismatches. A neighbor-joining tree was produced from this collection of genomes using Snippy (https://github.com/tseemann/snippy).

### Compositional analyses

#### α-Diversity

We calculated the inverse Simpson index from relative ASV abundances (*p*) with *N* ASVs in a given sample, Eq. (1):

1 $${IVS}=\frac{{{1}}}{{\sum }_{{{i}}}^{{{N}}}{{{p}}}_{{{i}}}^{{{2}}}}$$

#### Principal coordinate analyses

Bray–Curtis distances were calculated from the filtered ASV table using QIIME v1.9.1 and principal components of the resulting distance matrix were calculated using the scikit-learn v1.0.2 package for the Python programming language, used to embed sample compositions in the first two principal coordinates.

#### Average compositions and manipulation of compositions

To describe the average composition of a set of samples we calculated the central tendency of a compositional sample^89^. For counter factual statistical analyses that require changes to a composition, e.g., an increase in a specific taxon, we deployed the perturbation operation (⊕), which is the compositional analog to addition in Euclidean space^89^. A sample *x* containing the original relative taxon abundances is perturbed by a vector *y*, Eq. (2):

2 $${{y}}:{{x}}\,{{\oplus }}\,{{y}}{{=}}\left[\frac{{{{x}}}_{{{{{{\bf{1}}}}}}}{{{y}}}_{{{{{{\bf{1}}}}}}}}{{\sum}_{{{i}}{{=}}{{{{{\bf{1}}}}}}}^{{{D}}}{{{x}}}_{{{i}}}{{{y}}}_{{{i}}}}{{,}}\frac{{{{x}}}_{{{{{{\bf{2}}}}}}}{{{y}}}_{{{{{{\bf{2}}}}}}}}{{\sum }_{{{i}}{{=}}{{{{{\bf{1}}}}}}}^{{{D}}}{{{x}}}_{{{i}}}{{{y}}}_{{{i}}}}{{,}}{{\ldots }}{{,}}\frac{{{{x}}}_{{{D}}}{{{y}}}_{{{D}}}}{{\sum }_{{{i}}{{=}}{{{{{\bf{1}}}}}}}^{{{D}}}{{{x}}}_{{{i}}}{{{y}}}_{{{i}}}}\right]{{\forall }}{{x}}{{,}}\ {{y}}\;{{\in }}\;{{{S}}}^{{{D}}}$$

where *S*^*D*^ represents the D-part simplex.

#### Bayesian t-test

To compare diversity measurements between different sample groups, e.g., different clinical status, we performed a Bayesian estimation of group differences (BEST^90^), implemented using the pymc3 v3.11 package for the Python programming language; with priors (∼) and deterministic calculations (=) to assess differences in estimated group means as follows:

$${{{{{{\rm{g}}}}}}}_{1}\sim {{{{{\rm{Normal}}}}}}\left({{\mu }}=15,\ {{\sigma }}=15\right)$$

$${{{{{{\rm{g}}}}}}}_{2}\sim {{{{{\rm{Normal}}}}}}({{\mu }}=15,\ {{\sigma }}=15)$$

$${{{\sigma }}}_{{{{{{\rm{g}}}}}}1}\sim {{{{{\rm{Uniform}}}}}}({{{{{\rm{low}}}}}}=1{{{{{\rm{e}}}}}}-4,\ {{{{{\rm{high}}}}}}=30)$$

$${{{\sigma }}}_{{{{{{\rm{g}}}}}}2}\sim {{{{{\rm{Uniform}}}}}}({{{{{\rm{low}}}}}}=1{{{{{\rm{e}}}}}}-4,\ {{{{{\rm{high}}}}}}=30)$$

$${{\nu }}\sim {{{{{\rm{Exponential}}}}}}(1/15)+1$$

$${{{\lambda }}}_{1}={{{{\sigma }}}_{{{{{{\rm{g}}}}}}1}}^{-2}$$

$${{{\lambda }}}_{2}={{{{\sigma }}}_{{{{{{\rm{g}}}}}}2}}^{-2}$$

$${{{{{\rm{G}}}}}}1\sim {{{{{\rm{StudentT}}}}}}({{{{{\rm{nu}}}}}}={{\nu }},\ {{{{{\rm{mu}}}}}}={{{{{\rm{g}}}}}}1,\ {{{{{\rm{lam}}}}}}={{{\lambda }}}_{1})$$

$${{{{{\rm{G}}}}}}2\sim {{{{{\rm{StudentT}}}}}}({{{{{\rm{nu}}}}}}={{\nu }},\ {{{{{\rm{mu}}}}}}={{{{{\rm{g}}}}}}2,\ {{{{{\rm{lam}}}}}}={{{\lambda }}}_{2})$$

$$\triangle={{{{{\rm{G}}}}}}1-{{{{{\rm{G}}}}}}2$$

Bayesian inference was performed using “No U-turn sampling”^91^. Highest density intervals (HDI) of the posterior estimation of group differences (∆) were used to determine statistical certainty (***: 99% HDI > 0 or < 0, **: 95%HDI, *:90% HDI). The BEST code was implemented following the pymc3 documentation.

#### Cross-validated logistic regression to associate BSI cases with ASV composition

We first removed ASVs with low prevalence (present in fewer than 5% of all samples), and low abundances (maximum observed relative abundance < 0.01) leaving 269 ASVs. We then scaled the ASV relative abundances between 0 and 1 (min–max scaling) and performed logistic regressions, relating ASV abundances to BSI status (1: BSI, 0: non-BSI) using the scikit-learn v1.1 linear_model.LogisticRegressionCV module for the Python programming language with an L1 (lasso) penalty, iterating over a range of regularization strengths ([0.01, 0.1, 1, 10, 100, 1000]) using the “liblinear” solver. We retained the inferred ASV association coefficients with non-zero values for each tested regularization strength to visualize the cross-validation path.

#### Bayesian logistic regression

We performed a Bayesian logistic regression to distinguish compositional differences between infection-associated samples and samples from patients without secondary infections. We modeled the infection state of patient sample *i*, *y*_i_ with a Binomial likelihood:

$${{{y}}}_{{{{{{\rm{i}}}}}}}\sim {{{{{\rm{Binomial}}}}}}({{{{{\rm{n}}}}}}=1,\ {{{{{\rm{p}}}}}}={{{{{\rm{p}}}}}})$$

$${{{{{\rm{p}}}}}}={{{{{\rm{inverse\; logistic}}}}}}({{\alpha }}+{{{X}}}_{{{i}}}{{\beta }})$$

$${{\alpha }}\sim {{{{{\rm{Normal}}}}}}({{\mu }}=0,\,{{\sigma }}=1)$$

$${{\beta }}\sim {{{{{\rm{Normal}}}}}}({{\mu }}=0,\,{{\sigma }}=1)$$

Where prior distributions are indicated by ∼; *α* is the intercept of the generalized linear model, *β* is the coefficient vector for the log_10_-relative taxon abundances *X*_*i*_ in sample *i* or, in some cases, the binary indicator variable for gut microbiome domination.

### Reporting summary

Further information on research design is available in the Nature Research Reporting Summary linked to this article.

## Supplementary information

Supplementary Information

Description of Additional Supplementary files

Supplementary Data 1

Supplementary Data 2

Reporting Summary

## Data Availability

The raw sequencing data have been deposited on the Sequencing Reads Archive (SRA), and SRA accession numbers are available for two bioprojects corresponding to the mouse sequencing data PRJNA745367 (Supplementary Data 1, https://www.ncbi.nlm.nih.gov/bioproject/PRJNA745367) and the human stool samples PRJNA746322 (Supplementary Data 2, https://www.ncbi.nlm.nih.gov/bioproject/PRJNA746322). Databases/sets used in this study include SILVAv.138 (https://www.arb-silva.de/documentation/release-138/), Minikraken2 v2 (https://ccb.jhu.edu/software/kraken2/index.shtml), and all of the NCBI RefSeq assemblies as of 11/17/2021.

## Code availability

The relevant analyses were implemented in the R programming language, v4, freely available for download on https://www.r-project.org/, as well as in the Python programming language version 3.10, available for download on https://www.python.org/downloads/. The relevant packages used for analyses are listed in the corresponding methods sections.

## Bioethics statement

The collection of COVID-19 human biospecimens for research has been approved by the NYUSOM Institutional Review Board under il8-01121 Inflammatory Bowel Disease and Enteric Infection at NYU Langone Health. The data presented in this study were also approved by Yale Human Research Protection Program Institutional Review Boards (FWA00002571, protocol ID 2000027690). Informed consent was obtained from all enrolled patients.
