# Supplementary material for: Gut microbiome dysbiosis in antibiotic-treated COVID-19 patients is associated with microbial translocation and bacteremia
Source: Nat Commun. 2022 Nov 1;13:5926. doi: 10.1038/s41467-022-33395-6 (PMC9626559; doi:10.1038/s41467-022-33395-6)
Supplement: Supplementary file 1 — Supplementary Information [file 41467_2022_33395_MOESM1_ESM.pdf]

# Supplementary Information

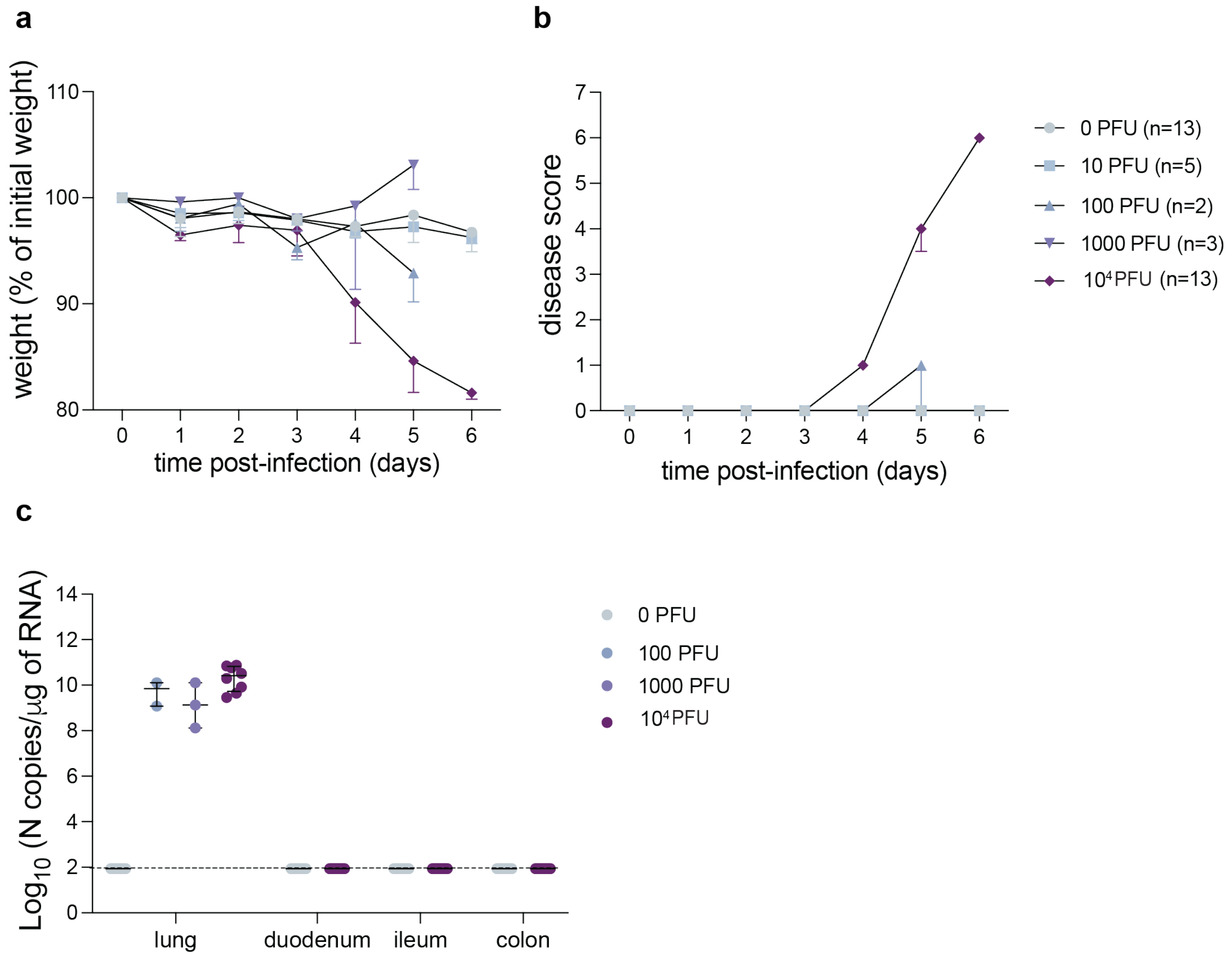


**Supplementary Fig. 1 SARS-CoV-2 infection in K18-hACE2 mice.**

**a-b** Following inoculation with 0, 10, 100, 1000 or $10^4$ PFU of SARS-CoV-2 or mock infection, mice were monitored daily for weight loss (a) and signs of disease quantified by a composite score based on ruffled fur, hunched back, heavy breathing and absence of mobility (b). Median and interquartile range determined for each group at each time point are depicted. Results are pooled from 1-3 independent experiments. For each group, the total number of mice is indicated. **c** Viral burden in lung or intestinal tissue of K18-hACE2 mice was analyzed at 5-6 days after infection with 100, 1000, $10^4$ PFU of SARS-CoV-2 or mock infection by qRT-PCR. Dots represent the copy number of N RNA per µg of RNA calculated for each mouse (n=13 for 0 PFU, 5 for 10 PFU, 2 for 100 PFU, 3 for 1000 PFU, and 13 for $10^4$ PFU). Results were pooled from 1 (100 and 1000 PFU doses) or 2 (mock and $10^4$ PFU) independent experiments with n=2-5 mice per group for each

experiment. The median and interquartile range are depicted for each experimental group. The dotted line depicts the limit of detection.

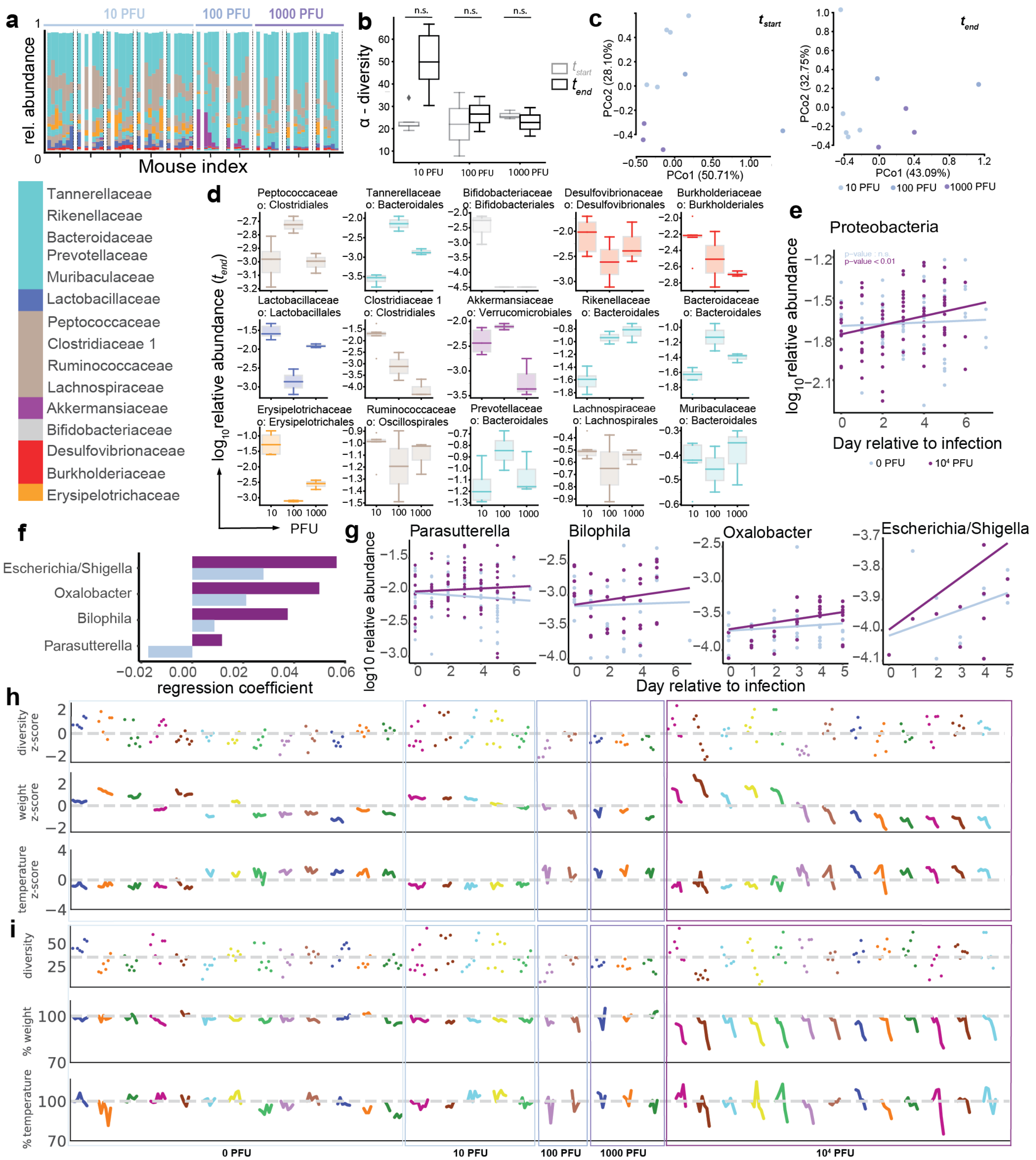
a
10 PFU
100 PFU
1000 PFU
rel. abundance
Mouse index
Tannerellaceae
Rikenellaceae
Bacteroidaceae
Prevotellaceae
Muribaculaceae
Lactobacillaceae
Peptococcaceae
Clostridiaceae 1
Ruminococcaceae
Lachnospiraceae
Akkermansiaceae
Bifidobacteriaceae
Desulfovibrionaceae
Burkholderiaceae
Erysipelotrichaceae
b
α - diversity
n.s.
$t_{start}$
$t_{end}$
10 PFU
100 PFU
1000 PFU
c
$t_{start}$
PCo2 (28.10%)
PCo1 (50.71%)
$t_{end}$
PCo2 (32.75%)
PCo1 (43.09%)
10 PFU
100 PFU
1000 PFU
d
$log_{10}$relative abundance ($t_{end}$)
Peptococcaceae o: Clostridiales
Tannerellaceae o: Bacteroidales
Bifidobacteriaceae o: Bifidobacteriales
Desulfovibrionaceae o: Desulfovibrionales
Burkholderiaceae o: Burkholderiales
Lactobacillaceae o: Lactobacillales
Clostridiaceae 1 o: Clostridiales
Akkermansiaceae o: Verrucomicrobiales
Rikenellaceae o: Bacteroidales
Bacteroidaceae o: Bacteroidales
Erysipelotrichaceae o: Erysipelotrichales
Ruminococcaceae o: Oscillospirales
Prevotellaceae o: Bacteroidales
Lachnospiraceae o: Lachnospirales
Muribaculaceae o: Bacteroidales
PFU
e
Proteobacteria
$log_{10}$relative abundance
p-value : n.s.
p-value < 0.01
Day relative to infection
0 PFU
$10^4$ PFU
f
Escherichia/Shigella
Oxalobacter
Bilophila
Parasutterella
regression coefficient
g
Parasutterella
Bilophila
Oxalobacter
Escherichia/Shigella
log10 relative abundance
Day relative to infection
h
diversity z-score
weight z-score
temperature z-score
i
diversity
% weight
% temperature
0 PFU
10 PFU
100 PFU
1000 PFU
$10^4$ PFU

**Supplementary Fig. 2 Inconsistent microbiomes dynamics in mice with lower infection doses.** **a** Bars represent bacterial family compositions in stool samples collected from each mouse over time, mouse time courses grouped as indicated by boxes. **b** Bacterial alpha diversity in first ($t_{start}$) and last ($t_{end}$) samples collected (n=5 for 10 PFU, 2 for 100 PFU, 3 for 1000 PFU; boxplots show median and quartile ranges). **c** principal coordinate plots of bacterial compositions in first and last samples colored by infection dose (in PFU). **d** Bacterial family abundances by infection dose at the final sample collected (n=5 for 10 PFU, 2 for 100 PFU, 3 for 1000 PFU; boxplots show median and quartile ranges). **e** $Log_{10}$ relative abundances of Proteobacteria in samples from $10^4$ PFU mice and uninfected; the lines show the maximum likelihood estimates of the inferred coefficients from a mixed effects time series model estimating Proteobacteria trajectories in the two experimental groups. **f** The maximum likelihood coefficient estimates for the four abundant Proteobacteria genera for the $10^4$ PFU mice and uninfected. **g** $Log_{10}$ relative abundances of the four genera, *Parasutterella*, *Bilophila*, *Oxalobacter* and *Escherichia/Shigella*; the lines show the regression lines of the time series model inferred from these data, visualizing the maximum likelihood coefficient estimates for time trajectories in the two experimental groups. **h** diversity, weight and temperature z-scores (calculated from all data points) over time per mouse as shown in a and Fig. 1. **i** untransformed diversity, weights and temperatures relative to the beginning of the experiment.

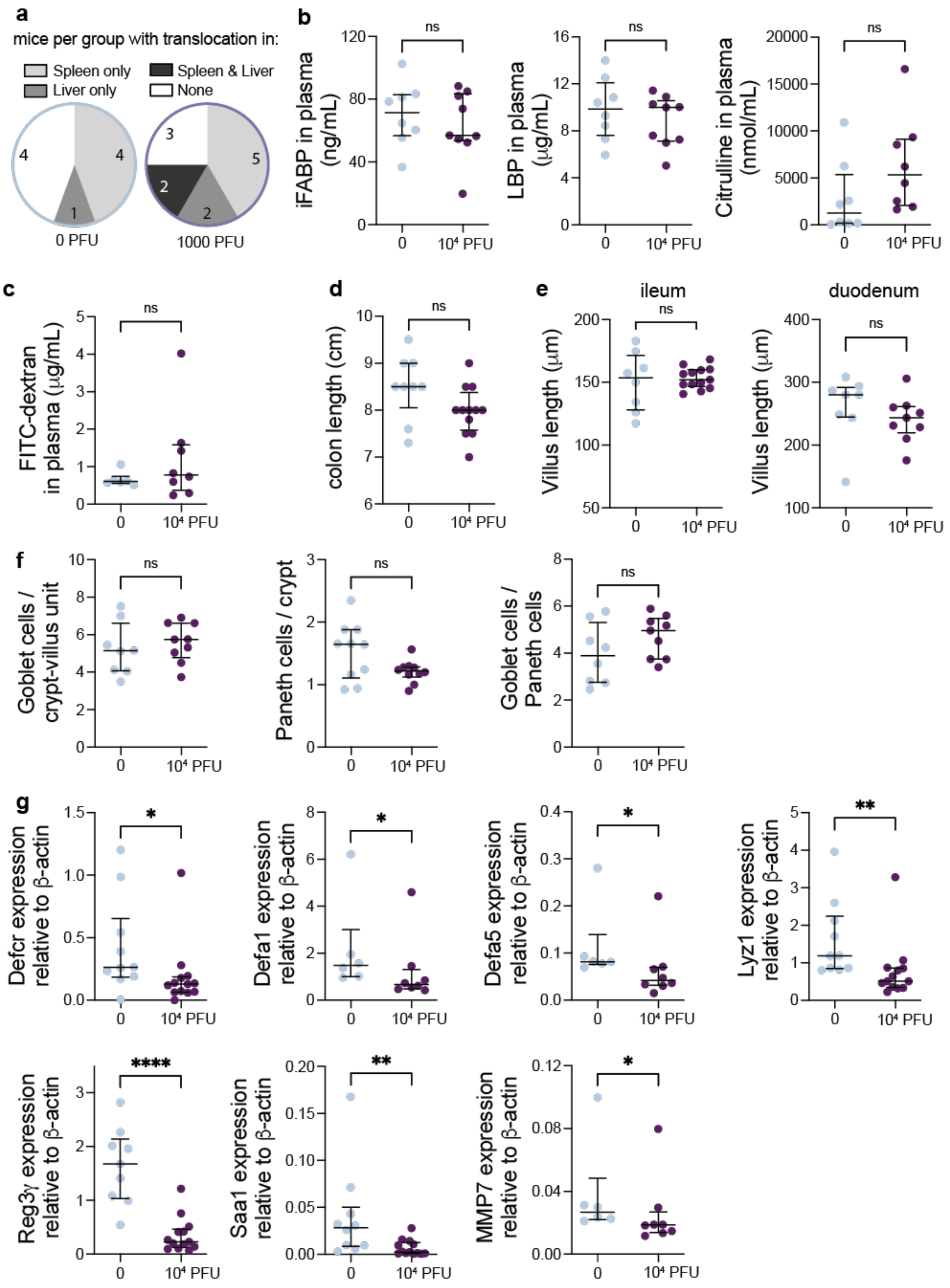

a
mice per group with translocation in:
Spleen only
Spleen & Liver
Liver only
None
0 PFU
1000 PFU
b
iFABP in plasma (ng/mL)
LBP in plasma (μg/mL)
Citrulline in plasma (nmol/mL)
ns
0
10⁴ PFU
c
FITC-dextran in plasma (μg/mL)
d
colon length (cm)
e
ileum
duodenum
Villus length (μm)
f
Goblet cells / crypt-villus unit
Paneth cells / crypt
Goblet cells / Paneth cells
g
Defcr expression relative to β-actin
Defa1 expression relative to β-actin
Defa5 expression relative to β-actin
Lyz1 expression relative to β-actin
Reg3γ expression relative to β-actin
Saa1 expression relative to β-actin
MMP7 expression relative to β-actin
*
**
****

**Supplementary Fig. 3 Characterization of the intestine during SARS-CoV-2 infection.** K18-hACE2 mice were inoculation with 1000 (a) or $10^4$ PFU SARS-CoV-2 (b-g) or PBS as mock treatment and analyzed at the end of the experiment. **a** Number of mice displaying bacteria in spleen, liver, or both organs as assessed by plating tissue homogenates on BBL™ Enterococcosel™ Agar, (BBL, modified esculin bile agar). All colonies were considered independently of their aspects. n=9 uninfected and 12 infected mice pooled from 2 independent experiments. The lower dose of 1000 PFU was used in this assay because mice inoculated with $10^4$ PFU were euthanized due to excessive morbidity before the pre-determined time point for organ collection. **b** Intestinal fatty acid-binding protein (iFABP), LPS-binding protein (LBP), and citrulline concentration in plasma at sacrifice. **c** Quantification of fluorescence intensity in the blood following oral administration of FITC-dextran. **d** Quantification of colon length. **e** Quantification of villus length in the duodenum (left) and ileum (right) based on H&E staining. **f** Quantification of goblet cell number (left) and Paneth cell number (middle) per crypt-villus unit in the proximal duodenum based on H&E staining and calculation of goblet cell per Paneth cell ratio based on these quantifications (right). **g** Relative expression of cryptidins (either using the pan-cryptidin primers $Defcr_{p130}$ and $Defcr_{m380}$ (Defcr) or specific primers for Defa1 and Defa5), lysozyme (Lyz1), matrix metallopeptidase 7 (MMP7), regenerating islet-derived protein 3-gamma (Reg3γ), and serum amyloid A (saa1) compared to β-actin in ileum homogenate as detected by qRT-PCR is depicted for each mouse. The black bar represents the median and interquartile range. Results were pooled from 2 or 3 independent experiments with n=3-5 mice per group for each experiment (n=6-10 control mice, 8-13 infected mice). Individual mice, represented by the circles as well as the median and interquartile ranges are depicted. In d, e, each circle shows the mean for each mouse of the cell number counted per crypt-villus unit on 50 units. Significant differences were determined using the two-sided Mann-Whitney U test (ns=non-significant, $p > 0.05$; **, $p < 0.01$; ***, $p < 0.001$; ****, $p < 0.0001$; boxplots show median and interquartile ranges).

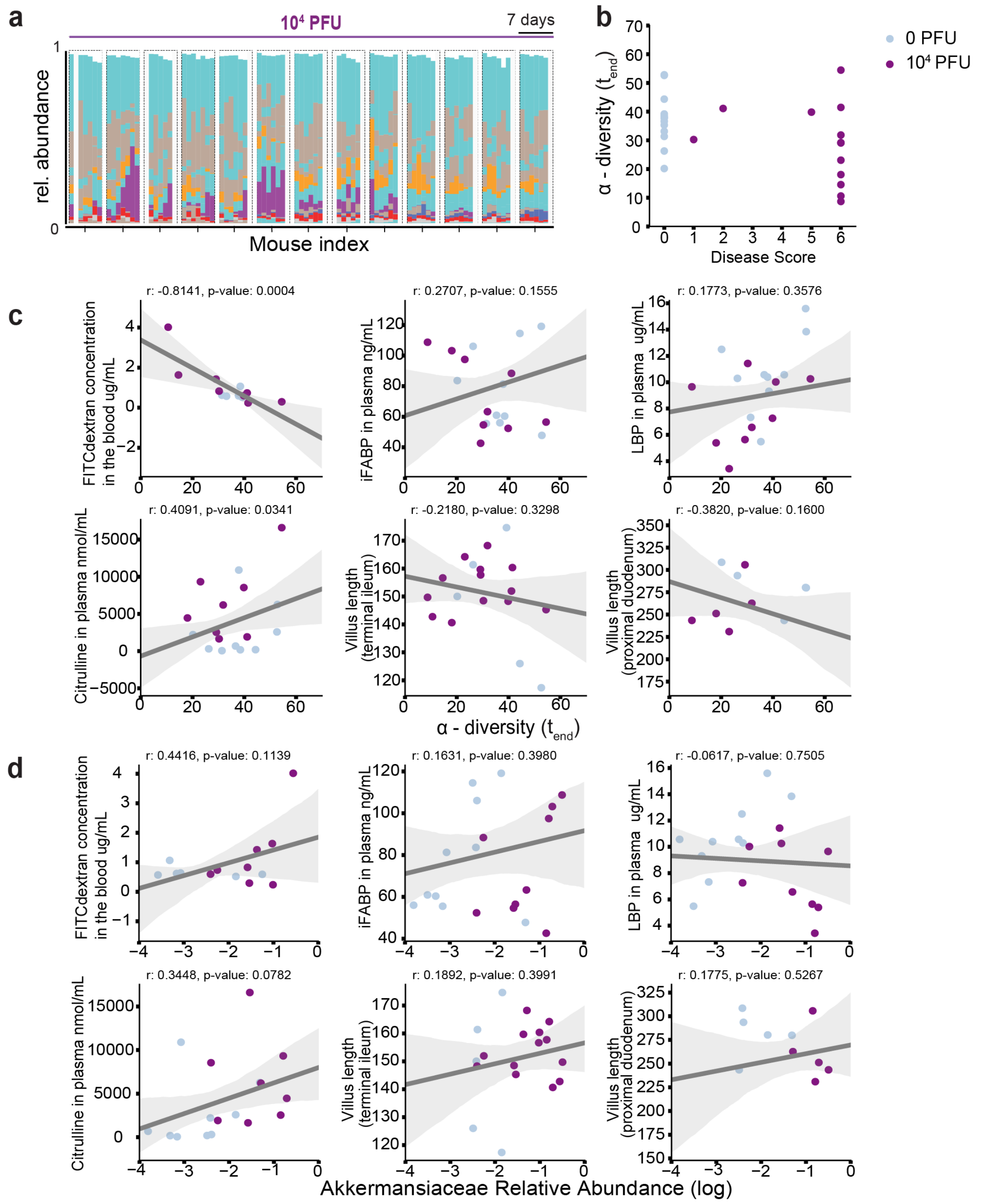


**Supplementary Fig. 4 Strongest gut dysbiosis is correlated with markers of defects in the intestinal barrier and epithelium. a** Reproduction of Fig. 1 showing bacterial compositions in mice infected with $10^4$ PFUs. **b** Disease scores vs diversity at the end of the experiment. **c-d** Correlations between alpha diversity (**c**) (inverse Simpson) and $\log_{10}$ relative *Akkermansia* abundances (**d**) at the end of the experiment with epithelium phenotypes and gut barrier integrity markers measured in the blood of mice (lines: linear regression, shaded region: 95%CI).

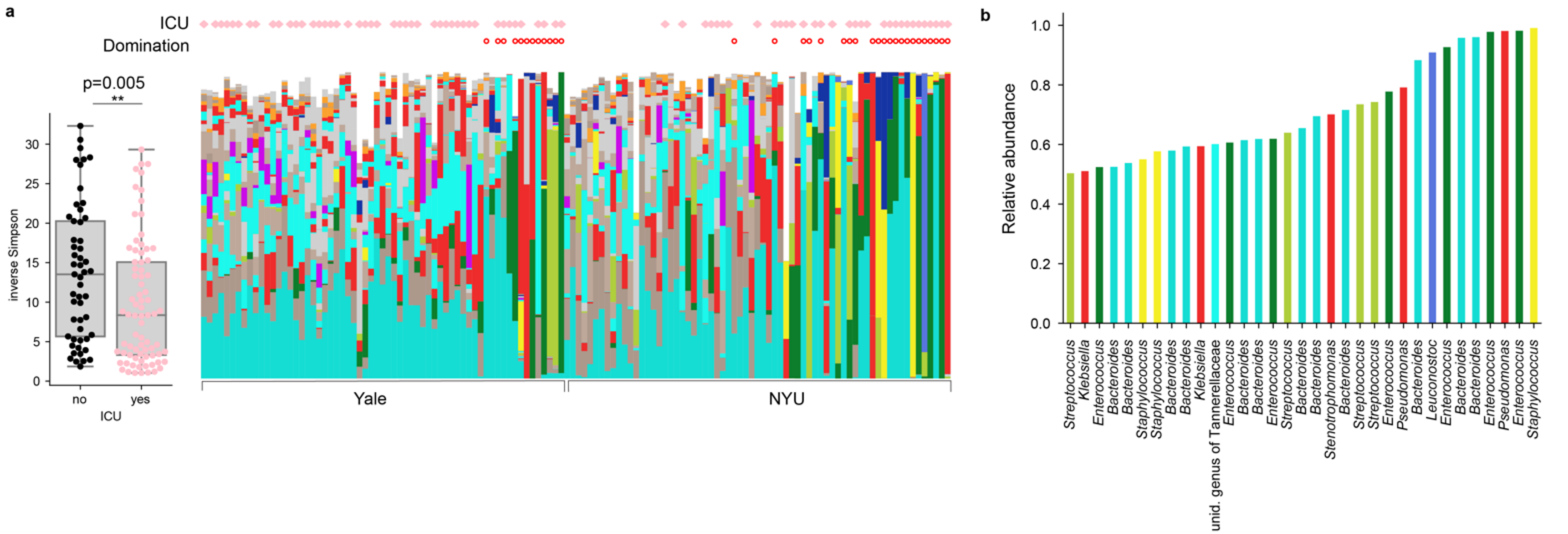


**Supplementary Fig. 5 a** Samples from patients requiring ICU transfer have lower diversity on average (n=130,p=0.005, two-sided Wilcoxon rank-sum; boxplots show median and quartile ranges); bars as in Fig. 1 with ICU status of patients and domination state of samples indicated. **b** Genus abundances in samples with a single genus >50% relative abundance.

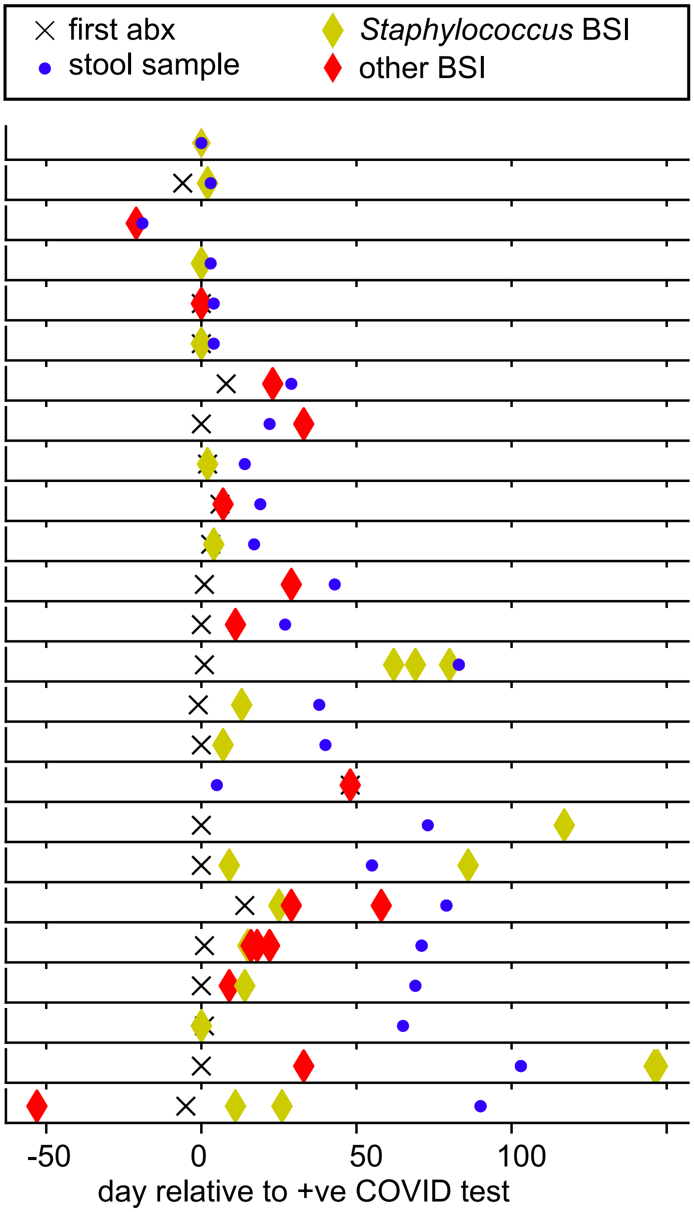


**Supplementary Fig. 6** Patients with a positive clinical blood culture result (BSI) received antibiotics, prior or on the day of blood culture results (cross symbol: first recorded antibiotic administration, blue: sequenced stool sample, diamond: positive blood culture result (BSI)).

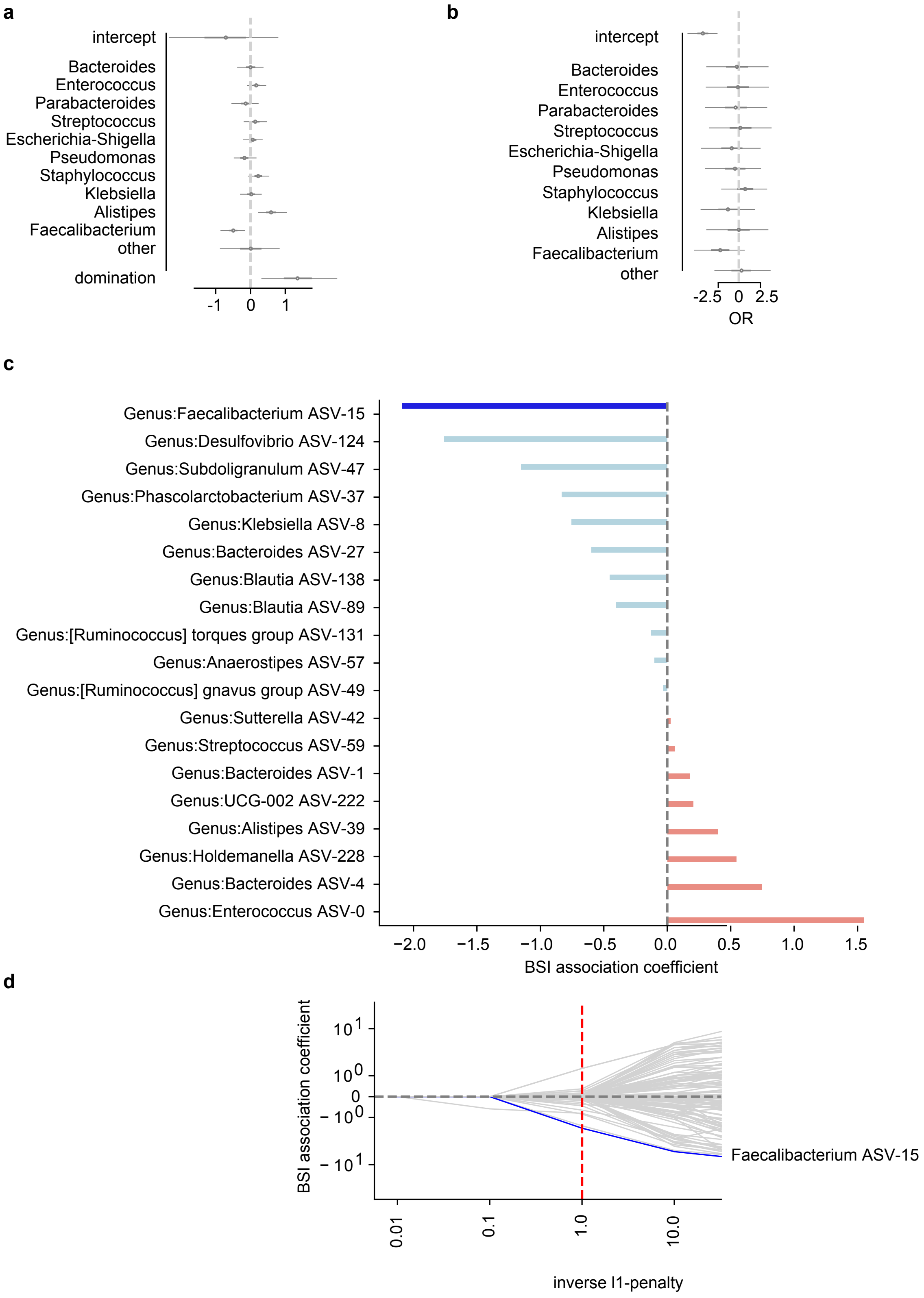
a
intercept
Bacteroides
Enterococcus
Parabacteroides
Streptococcus
Escherichia-Shigella
Pseudomonas
Staphylococcus
Klebsiella
Alistipes
Faecalibacterium
other
domination
-1
0
1
b
intercept
Bacteroides
Enterococcus
Parabacteroides
Streptococcus
Escherichia-Shigella
Pseudomonas
Staphylococcus
Klebsiella
Alistipes
Faecalibacterium
other
-2.5
0
2.5
OR
c
Genus:Faecalibacterium ASV-15
Genus:Desulfovibrio ASV-124
Genus:Subdoligranulum ASV-47
Genus:Phascolarctobacterium ASV-37
Genus:Klebsiella ASV-8
Genus:Bacteroides ASV-27
Genus:Blautia ASV-138
Genus:Blautia ASV-89
Genus:[Ruminococcus] torques group ASV-131
Genus:Anaerostipes ASV-57
Genus:[Ruminococcus] gnavus group ASV-49
Genus:Sutterella ASV-42
Genus:Streptococcus ASV-59
Genus:Bacteroides ASV-1
Genus:UCG-002 ASV-222
Genus:Alistipes ASV-39
Genus:Holdemanella ASV-228
Genus:Bacteroides ASV-4
Genus:Enterococcus ASV-0
−2.0
−1.5
−1.0
−0.5
0.0
0.5
1.0
1.5
BSI association coefficient
d
BSI association coefficient
$10^1$
$10^0$
0
$-10^0$
$-10^1$
Faecalibacterium ASV-15
0.01
0.1
1.0
10.0
inverse l1-penalty

**Supplementary Fig. 7 a** Posterior coefficient estimates from a Bayesian logistic regression regressing $\log_{10}$ relative abundances of the top 10 most abundant bacterial genera on BSI status using only BSI cases with associated stool samples taken prior or on the day of a confirmed positive blood culture (circle: posterior mean, lines: 95% HDI). **b** Posterior coefficient estimates from a Bayesian logistic regression regressing $\log_{10}$ relative abundances of the top 10 most abundant bacterial genera on BSI status with domination status of the microbiome as an additional predictor (domination: >50% of the composition by one taxon; circle: posterior mean, lines: 95% HDI). **c** ASVs associated with samples from patients with BSI. Coefficients from a cross-validated, L1-penalized logistic regression correlating the binary outcome (BSI) with $\log_{10}$-transformed relative ASV abundances. **d** Cross-validation paths; for all regularization strengths (L1-penalty) used, a *Faecalibacterium* ASV was most negatively associated with BSI-positive samples.

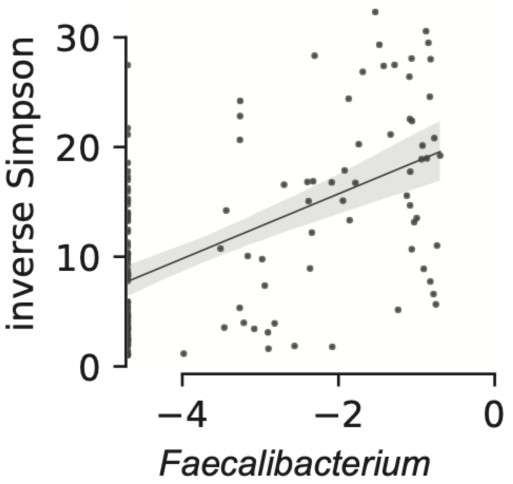


**Supplementary Fig. 8** ***Faecalibacterium*** **relative abundance is positively correlated with bacterial alpha diversity.** Log10 transformed relative abundances of the genus *Faecalibacterium* in stool samples from patients are correlated with the inverse Simpson diversity index; line from linear regression, shaded region: 95%CI.

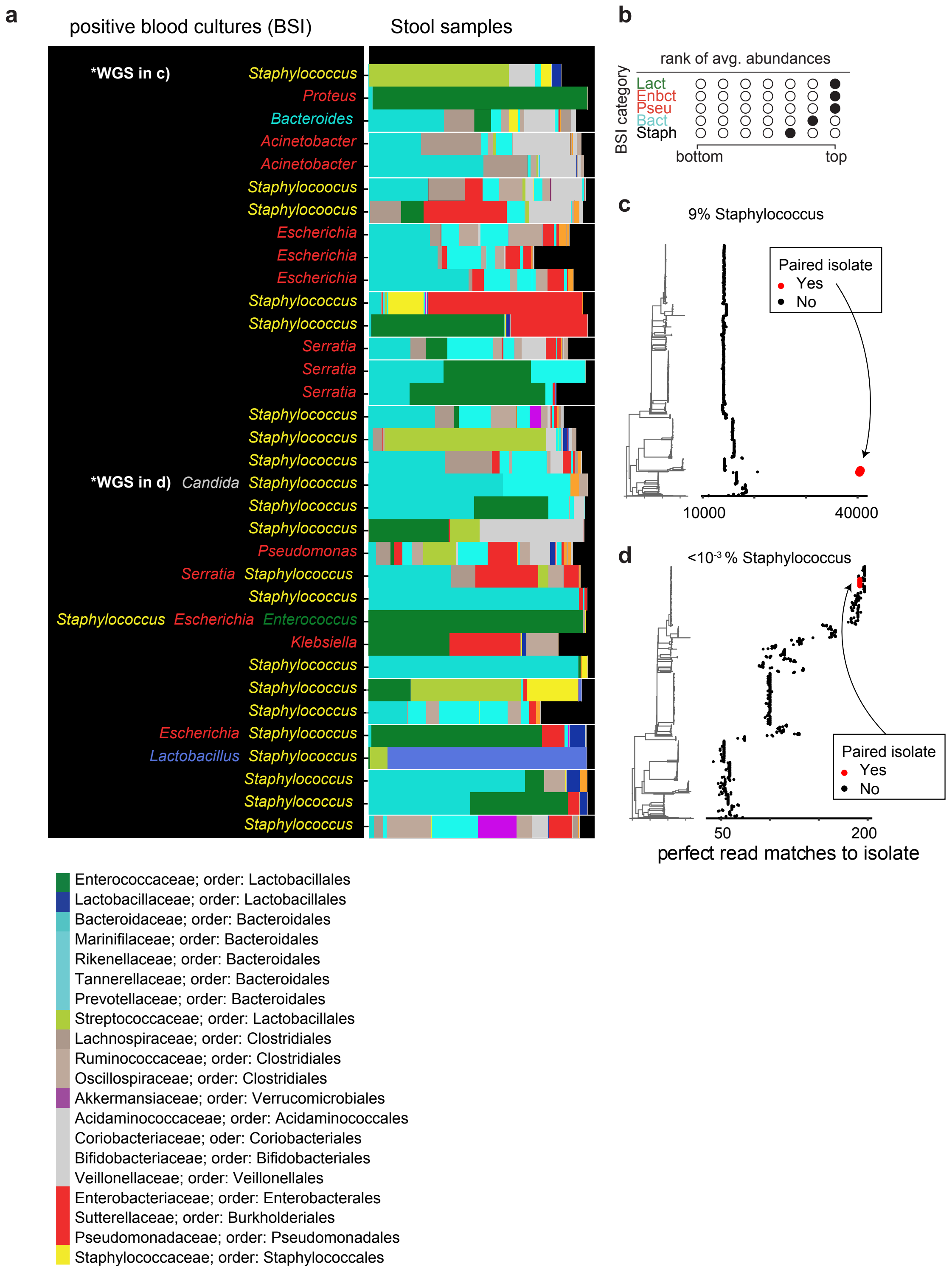
a
positive blood cultures (BSI)
Stool samples
*WGS in c)
Staphylococcus
Proteus
Bacteroides
Acinetobacter
Acinetobacter
Staphylococcus
Staphylococcus
Escherichia
Escherichia
Escherichia
Staphylococcus
Staphylococcus
Serratia
Serratia
Serratia
Staphylococcus
Staphylococcus
Staphylococcus
*WGS in d) Candida Staphylococcus
Staphylococcus
Staphylococcus
Pseudomonas
Serratia Staphylococcus
Staphylococcus
Staphylococcus Escherichia Enterococcus
Klebsiella
Staphylococcus
Staphylococcus
Staphylococcus
Escherichia Staphylococcus
Lactobacillus Staphylococcus
Staphylococcus
Staphylococcus
Staphylococcus
Enterococcaceae; order: Lactobacillales
Lactobacillaceae; order: Lactobacillales
Bacteroidaceae; order: Bacteroidales
Marinifilaceae; order: Bacteroidales
Rikenellaceae; order: Bacteroidales
Tannerellaceae; order: Bacteroidales
Prevotellaceae; order: Bacteroidales
Streptococcaceae; order: Lactobacillales
Lachnospiraceae; order: Clostridiales
Ruminococcaceae; order: Clostridiales
Oscillospiraceae; order: Clostridiales
Akkermansiaceae; order: Verrucomicrobiales
Acidaminococcaceae; order: Acidaminococcales
Coriobacteriaceae; oder: Coriobacteriales
Bifidobacteriaceae; order: Bifidobacteriales
Veillonellaceae; order: Veillonellales
Enterobacteriaceae; order: Enterobacterales
Sutterellaceae; order: Burkholderiales
Pseudomonadaceae; order: Pseudomonadales
Staphylococcaceae; order: Staphylococcales
b
rank of avg. abundances
BSI category
Lact
Enbct
Pseu
Bact
Staph
bottom
top
c
9% Staphylococcus
Paired isolate
Yes
No
10000
40000
d
<10^-3 % Staphylococcus
Paired isolate
Yes
No
50
200
perfect read matches to isolate

**Supplementary Fig. 9 Bacteria in stool of COVID-19 patients match taxa identified blood cultures. a** Organisms identified in blood cultures together with bars representing the bacterial family compositions in stool samples; multiple samples belonging to the same patient grouped by a white box. Two samples with matching whole genome sequenced (WGS) blood isolates indicated. **b** Rank analysis of abundance patterns in stool samples from different BSI categories; a filled circle indicates the calculated rank of the focal BSI category (row) in terms of the corresponding taxon stool abundance relative to samples from other BSI categories (Lact: Lactobacillales, Enbct: Enterobacterales; Pseu: Pseudomonadales, Bact: Bacteroidales, Staph: Staphylococcales. Only 5 out of 7 BSI categories are shown because fungal BSIs and the uninfected category have no corresponding bacterial stool abundances). **c,d** left: neighbor-joining tree constructed from all NCBI RefSeq assemblies of *Staphylococcus aureus* genomes in addition to isolates that were isolated from subjects highlighted in **a**. right: counts of perfect read matches of shotgun metagenomic reads from stool samples, red: stool sample sequencing read matches to WGS of isolates from the same patient, black: matches to other genomes.

**Supplementary Table 1: Clinical characteristics of patients with confirmed COVID-19 at NYU Langone Health and Yale New Haven Hospital**

| | **NYU, N = 60** | **YALE, N = 36** |
|---|---|---|
| Age (years) | 51 ± 17.5 | 62.52 ± 19.72 |
| Sex (F \| M) | 42% \| 58% | 39% \| 61% |
| **Hospital course and Outcomes** | | |
| ICU Admission | 53% | 65% |
| Pneumonia | 42% | 77% |
| Diarrhea | 13% | 32% |
| Intubation | 36% | 41% |
| Sepsis | 23% | 18% |
| Encephalopathy | 12% | 3% |
| Death | 5% | 21% |
| Length of stay (median, IQR) | 37 (10-86) | 27 (11-35.25) |
| **Risk Factors** | | |
| Cancer within 1 year | 7% | 4% |
| Chronic Heart Disease | 18% | 36% |
| Hypertension | 38% | 64% |
| Chronic Lung Disease | 7% | 20% |
| Immunosuppression | 17% | 4% |

**Supplementary Table 2: Clinical characteristics of COVID-19 patients at NYU Langone Health and Yale New Haven Hospital with and without positive blood culture results (BSI).**

| | **BSI, N = 26** | **non-BSI N = 53** |
|---|---|---|
| **Hospital course and Outcomes** | | |
| ICU Admission | 69% | 64% |
| Pneumonia | 73% | 53% |
| Diarrhea | 31% | 64% |
| Intubation | 58% | 36% |
| Sepsis | 35% | 21% |
| Encephalopathy | 19% | 6% |
| Death | 15% | 9% |
| Length of stay (median, IQR) | 59 (23-91.5) | 22 (6-51) |

**Supplementary Table 3: Shotgun metagenomic reads mapped to species identified in clinical blood cultures**. Dark grey shading: no sequencing reads from stool samples matched the species identified in clinical blood samples, light grey shading: species of the same genus but not the same species had non-zero read counts in stool samples. The relative abundance of identified species were contrasted with their mean abundances (log10 ratio).

| **Organism identified in blood** | **species identified in stool sample** | **Log ratio** |
|---|---|---|
| *Bacteroides thetaiotaomicron* | Bacteroides_thetaiotaomicron_14-106904-2 | 2.92 |
| *Enterococcus faecalis* Group D | Enterococcus_faecalis_LD33 | 1.8 |
| *Escherichia coli* | Escherichia_coli_K-12_substr._W3110 | 2.2 |
| *Escherichia coli* | Escherichia_coli_IAI39 | 1.6 |
| *Escherichia coli* | Escherichia_coli_536 | 2.8 |
| *Klebsiella pneumoniae* | Klebsiella_pneumoniae_KPNIH27 | -1.7 |
| *Lactobacillus species* | Lactobacillus_curvatus_WiKim38 | 3.9 |
| *Pseudomonas aeruginosa* | Pseudomonas_aeruginosa_SJTD-1 | 3.3 |
| *Serratia marcescens* | Serratia_marcescens_CAV1492 | -0.2 |
| *Staphylococcus aureus* | Staphylococcus_aureus_RF122 | 1.9 |
| *Proteus mirabilis* | Proteus_mirabilis;t__Proteus_mirabilis_BB2000 | 0.56 |
| *Acinetobacter lwolfii* | Acinetobacter_calcoaceticus_EGD_AQ_BF14 | -0.6 |
| *Staphylococcus* | Staphylococcus_sp._HMSC063G01_HMSC063G01 | 0.9 |
| *Staphylococcus* | Staphylococcus_epidermidis_W23144 | 3.3 |
| *Staphylococcus aureus* | not found | |
| *Staphylococcus hominis* | not found | |
| *Staphylococcus capitis* | not found | |
| *Staphylococcus epidermidis*, *hominis* | Staphylococcus_pseudintermedius_063228 | 2.2 |
| *Staphylococcus epidermidis*, *hominis* ssp hominis | not found | |
| *Staphylococcus epidermidis* | Staphylococcus_aureus_JKD6008 | 2.7 |
| *Staphylococcus epidermidis* | Staphylococcus_epidermidis_DAR1907 | 1.1 |
| *Staphylococcus capitis* | Staphylococcus_sp._HMSC067F07_HMSC067F07 | 3.7 |
| *Staphylococcus epidermidis*, hominis ssp hominis | Staphylococcus_hominis_793_SHAE | 0.8 |
| *Staphylococcus epidermidis* | Staphylococcus_sp._HMSC070D05_HMSC070D05 | 3.8 |
| *Staphylococcus hominis*, *epidermidis* | Staphylococcus_hominis_MMP2 | 1.0 |
| *Staphylococcus hominis*, *epidermidis* | Staphylococcus_epidermidis_ATCC12228 GCF7645.1 | 0.2 |

**Supplementary Table 4: Primers used for qRT-PCR analysis of antimicrobial factors.**

| Gene | Forward Primer (5'-3') | Reverse Primer (5'-3') | Source |
|---|---|---|---|
| *β-actin* | CGGTTCCGATGCCCTGAGGCTCTT | CGTCACACTTCATGATGGAATTGA | PMID: 31284554 |
| *Defcr* | Defcrp130-<br>AAGAGACTAAAACTGAGGAGCAGC | Defcrm380-<br>GGTGATCATCAGACCCCAGCATCAGT | PMID: 9038894 |
| *Defa1* | TCCTCCTCTCTGCCCTTGTC | CCTTTGCAGCCTCTTGATCT | PMID: 33154750 |
| *Defa5* | TCAAAAAAGCTGATATGCTATTG | AGCTGCAGCAGAATACGAAAG | PMID: 31511628 |
| *Lyz1* | TTCGAGCATGGGTGGCATGG | GGCTGCAGTAGAAGCACACC | PMID: 22137268 |
| *MMP7* | TTCAAGAGGGTTAGTTGGGGGACTG | TTGTCAAAGTGAGCATCTCCGCC | PMID 18637162 |
| *Reg3γ* | CGTGCCTATGGCTCCTATTGCT | TTCAGCGCCACTGAGCACAGAC | PMID: 24096422 |
| *SAA1* | CATTTGTTCACGAGGCTTTCC | GTTTTTCCAGTTAGCTTCC TTCATGT | PMID: 25073702 |
