# Supplementary material for: Gut microbiome dysbiosis in antibiotic-treated COVID-19 patients is associated with microbial translocation and bacteremia
Source: Nat Commun. 2022 Nov 1;13:5926. doi: 10.1038/s41467-022-33395-6 (PMC9626559; doi:10.1038/s41467-022-33395-6)
Supplement: Supplementary file 2 — Description of Additional Supplementary files [file 41467_2022_33395_MOESM2_ESM.pdf]

Description of Additional Supplementary Files

Supplementary Data 1: SRA accession numbers for the bioproject PRJNA745367 corresponding to the mouse sequencing data.

Supplementary Data 2: SRA accession numbers for the bioproject PRJNA746322 corresponding to the human stool samples sequencing data.
