# Supplementary material for: Gut microbiome dysbiosis in antibiotic-treated COVID-19 patients is associated with microbial translocation and bacteremia
Source: Nat Commun. 2022 Nov 1;13:5926. doi: 10.1038/s41467-022-33395-6 (PMC9626559; doi:10.1038/s41467-022-33395-6)
Supplement: Supplementary file 5 — Reporting Summary [file 41467_2022_33395_MOESM5_ESM.pdf]

nature portfolio

Corresponding author(s): Ken Cadwell, Jonas Schluter

Last updated by author(s): Aug 27, 2022

Reporting Summary

Nature Portfolio wishes to improve the reproducibility of the work that we publish. This form provides structure for consistency and transparency in reporting. For further information on Nature Portfolio policies, see our Editorial Policies and the Editorial Policy Checklist.

Statistics

For all statistical analyses, confirm that the following items are present in the figure legend, table legend, main text, or Methods section.

n/a	Confirmed	
☐	☒	The exact sample size (*n*) for each experimental group/condition, given as a discrete number and unit of measurement
☒	☐	A statement on whether measurements were taken from distinct samples or whether the same sample was measured repeatedly
☐	☒	The statistical test(s) used AND whether they are one- or two-sided<br>*Only common tests should be described solely by name; describe more complex techniques in the Methods section.*
☐	☒	A description of all covariates tested
☐	☒	A description of any assumptions or corrections, such as tests of normality and adjustment for multiple comparisons
☐	☒	A full description of the statistical parameters including central tendency (e.g. means) or other basic estimates (e.g. regression coefficient) AND variation (e.g. standard deviation) or associated estimates of uncertainty (e.g. confidence intervals)
☐	☒	For null hypothesis testing, the test statistic (e.g. *F*, *t*, *r*) with confidence intervals, effect sizes, degrees of freedom and *P* value noted<br>*Give P values as exact values whenever suitable.*
☐	☒	For Bayesian analysis, information on the choice of priors and Markov chain Monte Carlo settings
☐	☒	For hierarchical and complex designs, identification of the appropriate level for tests and full reporting of outcomes
☒	☐	Estimates of effect sizes (e.g. Cohen's *d*, Pearson's *r*), indicating how they were calculated

Our web collection on statistics for biologists contains articles on many of the points above.

Software and code

Policy information about availability of computer code

Data collection	The clinical data were collected using EPIC EHR and REDCap 9.3.6 software.
Data analysis	The relevant analyses were implemented in the R programming language, v4, freely available for download on https://www.r-project.org/, as well as in the Python programming language version 3.10, available for download on https://www.python.org/downloads/. The relevant packages used for analyses are listed in the corresponding methods sections.<br><br>Time series analyses of bacterial family abundances: lmerTest v.3.1-3<br>Bioinformatic processing and taxonomic assignment: dada2 v1.16.0<br>Shotgun metagenomic sequencing: cutadapt, shi7, MetaPhlAn2<br>Mapping shotgun reads to whole genome sequences of clinical isolates: Kraken2, Bowtie2, Snippy<br>Principal coordinate analyses: QIIME v1.9.1, scikit-learn v1.0.2<br>Bayesian t-test: pymc3<br>Cross-validated logistic regression to associate BSI cases with ASV composition: scikit v1.1 sklearn.linear_model.LogisticRegressionCV, “liblinear” solver

For manuscripts utilizing custom algorithms or software that are central to the research but not yet described in published literature, software must be made available to editors and reviewers. We strongly encourage code deposition in a community repository (e.g. GitHub). See the Nature Portfolio guidelines for submitting code & software for further information.

Data

Policy information about availability of data

All manuscripts must include a data availability statement. This statement should provide the following information, where applicable:

- Accession codes, unique identifiers, or web links for publicly available datasets
- A description of any restrictions on data availability
- For clinical datasets or third party data, please ensure that the statement adheres to our policy

The raw sequencing data have been deposited on the Sequencing Reads Archive (SRA), and SRA accession numbers are available for two bioprojects corresponding to the mouse sequencing data PRJNA745367 (Supplementary Table 5) and the human stool samples PRJNA746322 (Supplementary Table 6). Databases/sets used in this study include SILVAv.138 (https://www.arb-silva.de/documentation/release-138/), Minikraken2 v2 (https://ccb.jhu.edu/software/kraken2/index.shtml), and all of the NCBI RefSeq assemblies as of 11/17/2021.

Human research participants

Policy information about studies involving human research participants and Sex and Gender in Research.

Reporting on sex and gender	Patient sex is reported in Supplementary Table 1. No sex-based analyses were performed.
Population characteristics	Population characteristics are provided in the Supplementary Tables 1 and 2.
Recruitment	This study involved 96 patients with laboratory-confirmed SARS-CoV-2 infection. SARS-CoV-2 infection was confirmed by a positive result of real-time reverse transcriptase-polymerase chain reaction assay on a nasopharyngeal swab. 60 patients were seen at NYU Langone Health, New York, between January 29, 2020 – July 2, 2020. In order to be eligible for inclusion in the study, stool specimens needed to be from individuals >18 years of age. In parallel, 36 patients were admitted to YNHH with COVID-19 between 18 March 2020 and 27 May 2020 as part of the YALE IMPACT cohort described at length elsewhere (Lucas et al 2020 Nature).<br><br>Only potential bias is location: Yale New Haven Hospital or NYU Langone Health.
Ethics oversight	The collection of COVID-19 human biospecimens for research has been approved by the NYUSOM Institutional Review Board under il8-01121 Inflammatory Bowel Disease and Enteric Infection at NYU Langone Health. The data presented in this study were also approved by Yale Human Research Protection Program Institutional Review Boards (FWA00002571, protocol ID 2000027690). Informed consent was obtained from all enrolled patients.

Note that full information on the approval of the study protocol must also be provided in the manuscript.

Field-specific reporting

Please select the one below that is the best fit for your research. If you are not sure, read the appropriate sections before making your selection.

☒ Life sciences ☐ Behavioural & social sciences ☐ Ecological, evolutionary & environmental sciences

For a reference copy of the document with all sections, see nature.com/documents/nr-reporting-summary-flat.pdf

Life sciences study design

All studies must disclose on these points even when the disclosure is negative.

Sample size	Sample sizes were not determined before the study. Human samples were collected as they became available. The number of animals used in the experiments in this study is estimated based on a power analysis with the following assumptions: standard<br>deviation will be ~20% of the mean, p-value will be under 0.05 when the null hypothesis is false, the effect size (Cohen's d) is between 1.0-2.0. The sample size was further informed by empirical evidence of what is necessary for interpretation of the data and statistical significance.
Data exclusions	Samples from non-adults (<18 years old) were excluded. Regarding mouse experiments, all samples were included except for the histology analysis when slides were not of sufficient quality for analysis.
Replication	This study involved 96 patients with laboratory-confirmed SARS-CoV-2 infection.<br>Mouse experiments were repeated 3 times, no difference was observed between the replicates.

Randomization	Human study: The experiments were not randomized; the Investigators were not blinded to allocation during experiments and outcome assessment.<br><br>Animals from the same breeder pool (i.e., littermates) were randomly assigned and housed in cages according to the experimental groups in ventilated racks and provided autoclaved water and standard chow ad libitum (dark/light cycle: 12/12 hours, ambient temperature: 69-72°F, humidity: 30-70%). Cage bedding was mixed prior to infection in a subset of experiments to further reduce possible cage effect.
Blinding	Human study: The experiments were not randomized; the Investigators were not blinded to allocation during experiments and outcome assessment.<br><br>Blinding was not used for the mice experiments due to restrained access to the BSL3 facility.

Reporting for specific materials, systems and methods

We require information from authors about some types of materials, experimental systems and methods used in many studies. Here, indicate whether each material, system or method listed is relevant to your study. If you are not sure if a list item applies to your research, read the appropriate section before selecting a response.

Materials & experimental systems

n/a	Involved in the study
☐	☒ Antibodies
☐	☒ Eukaryotic cell lines
☒	☐ Palaeontology and archaeology
☐	☒ Animals and other organisms
☐	☒ Clinical data
☒	☐ Dual use research of concern

Methods

n/a	Involved in the study
☒	☐ ChIP-seq
☒	☐ Flow cytometry
☒	☐ MRI-based neuroimaging

Antibodies

Antibodies used	anti-lysozyme ab108508, Abcam
Validation	Recombinant Anti-Lysozyme andibody ab108508 [EPR2994(2)] is a rabbit monoclonal antibody which detects lysozyme protein of mouse and human. Application: immunohistochemistry, western blot. Citation: Yagishita Y et al. Cell Mol Gastroenterol Hepatol 11:503-524 (2021).<br><br>The antibody was knockout validated by the manufacturer and in house-validated by our microscopy core. Dilution 1:1000.

Eukaryotic cell lines

Policy information about cell lines and Sex and Gender in Research

Cell line source(s)	Vero E6 (CRL-1586) were obtained from the American Type Culture Collection
Authentication	N/A. The cell line was not authenticated because it was used for viral propagation and not for generating data points.
Mycoplasma contamination	The cell line was not tested for Mycoplasma contamination
Commonly misidentified lines (See ICLAC register)	No commonly misidentified cell lines were used in the study.

Animals and other research organisms

Policy information about studies involving animals; ARRIVE guidelines recommended for reporting animal research, and Sex and Gender in Research

Laboratory animals	Heterozygous K18-hACE2 C57BL/6J mice (strain: 2B6.Cg-Tg(K18-ACE2)2Prlmn/J) were obtained from The Jackson Laboratory. Several were paired with C57BL/6J mice to generate additional heterozygous mice for subsequent experiments and the remaining were used to perform initial experiments.<br><br>Animals from the same breeder pool (i.e., littermates) were randomly assigned and housed in cages according to the experimental groups in ventilated racks and provided autoclaved water and standard chow ad libitum (dark/light cycle: 12/12 hours, ambient temperature: 69-72°F, humidity: 30-70%).<br><br>12-week-old or 24-week-old K18-hACE2 males were administered either 10-10000 PFU SARS-CoV-2 diluted in 50µL PBS (Corning) or 50µL PBS (non-infected, 0) via intranasal administration under xylazine-ketamine anesthesia (AnaSedR AKORN Animal Health,

	KetathesiaTM Henry Schein Inc).
Wild animals	The study did not involve wild animals.
Reporting on sex	Only male mice were used in this study. This sex was chosen so that results could be compared with other models used in our lab, which indicated that male mice are more likely to survive long enough to allow for the assays we employed. Results observed could be different in female mice but as animal study in our BSL3 facility was challenging the number of mice had to be kept low.
Field-collected samples	The study did not include samples collected from the field.
Ethics oversight	All animal studies were performed according to protocols approved by the NYU School of Medicine Institutional Animal Care and Use Committee (IACUC n°170209 and 180802) and in the ABSL3 facility of NYU Grossman School of Medicine (New York, NY), in accordance with its Biosafety Manual and Standard Operating Procedures.

Note that full information on the approval of the study protocol must also be provided in the manuscript.

Clinical data

Policy information about clinical studies

All manuscripts should comply with the ICMJEguidelines for publication of clinical research and a completedCONSORT checklist must be included with all submissions.

Clinical trial registration	n/a
Study protocol	n/a
Data collection	Demographic information of patients was aggregated through a systematic and retrospective review of the EHR and was used to construct Supplementary Table 1. Symptom onset and etiology were recorded through standardized interviews with patients or patient surrogates upon enrollment in our study, or alternatively through manual EHR review if no interview was possible owing to clinical status at enrollment. The clinical data were collected using EPIC EHR and REDCap 9.3.6 software. At the time of sample acquisition and processing, investigators were blinded to patient clinical status.
Outcomes	n/a
